# Microalgae-Based Wastewater Treatment and Biomass Valorization: Insights, Challenges, and Opportunities from 15 Years of Research

**DOI:** 10.1021/acsomega.5c04364

**Published:** 2025-10-17

**Authors:** Maria Lúcia Calijuri, Eduardo de Aguiar Do Couto, Paula Peixoto Assemany, Vinicius José Ribeiro, Juliana Ferreira Lorentz, Jackeline de Siqueira Castro, Letícia Rodrigues de Assis, Adriana Paulo de Sousa Oliveira, Alexia Saleme Aona de Paula Pereira, Bianca Barros Marangon, Iara Barbosa Magalhães, Thiago Abrantes Silva, Jéssica Ferreira, Matheus Quintão Braga, Rafael Carvalho Nogueira da Gama, Bruno Silva Henriques

**Affiliations:** † Post-Graduate Programm in Civil Engineering, Center for Advanced Research in Microalgae, 28120 Federal University of Viçosa (universidade Federal de Viçosa), University Campus, Viçosa, Minas Gerais 36.570-900, Brazil; ‡ Post-Graduate Programm in Environmental Engineering, Environmental Engineering Department, Federal University of Lavras (universidade Federal de Lavras), University Campus, Lavras, Minas Gerais 37.203-202, Brazil

## Abstract

This study highlights 15 years of research conducted at the Federal University of Viçosa, Brazil, on utilizing microalgae for wastewater treatment and biomass valorization within the biorefinery framework. High-rate algal ponds (HRAPs) have emerged as an accessible, efficient, and sustainable technology for decentralized sanitation, particularly in remote areas. The research findings, consistent with global studies, demonstrate that strategies such as ultraviolet (UV) predisinfection and carbon dioxide (CO_2_) supplementation significantly enhance the removal of nitrogen (N), phosphorus (P), and organic matter while optimizing biomass productivity. Integrating domestic and industrial wastewater improved nutrient balance, while hybrid systems combining HRAPs with biofilm reactors further increased biomass yields and reduced operational costs. The resulting biomass exhibited high versatility, with proteins (32–42%) as the dominant fraction, followed by carbohydrates (18–23%) and lipids (13–16%), underscoring its potential for bioenergy and agricultural applications. Energy conversion pathways such as anaerobic digestion and hydrothermal liquefaction (HTL) effectively transformed wet biomass into biogas and bio-oil. However, challenges such as high ash and nitrogen content remain to be addressed. In agriculture, microalgae-derived biofertilizers enhanced soil health, reduced nutrient losses, and boosted crop productivity, demonstrating performance comparable to chemical fertilizers. Despite economic and environmental challenges, such as high drying costs and energy demands, the study underscores the significant potential of microalgae-based solutions in advancing the circular bioeconomy. This approach aligns with the Sustainable Development Goals (SDGs) by integrating wastewater treatment, clean energy production, and food security into a cohesive, sustainable framework.

## Graphical abstract

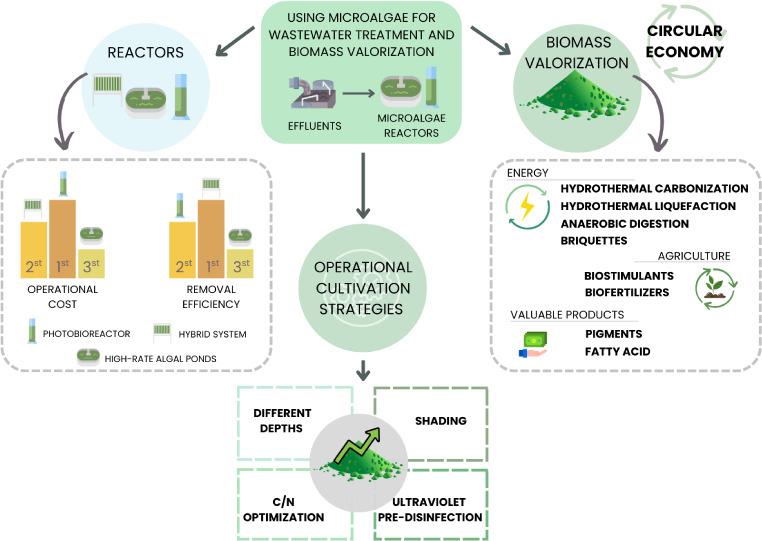

## 1 Introduction

Environmental challenges such as water pollution and climate change pose significant threats to society, leading to widespread social and economic consequences on a global scale. In 2015, the United Nations (UN) introduced 17 Sustainable Development Goals (SDGs) to be achieved by 2030, aiming to create a cleaner, more sustainable, and equitable society. However, substantial progress is still required to guarantee equal human rights, particularly in developing countries. Alarmingly, as of 2022, 3.5 billion people worldwide still lacked access to basic sanitation services.^1^ For instance, in Brazil, only 49.1% of the wastewater generated receives any treatment.^2^ It is estimated that approximately 1.7 billion dollars would be needed between 2016 and 2030 to achieve universal access to clean water, sanitation, and hygiene in 140 low- and middle-income countries.^3^ Considering the scale of the global economy, this amount is relatively modest. This realization highlights that the barriers to universal access extend beyond financial limitations to include political and technological challenges, particularly regarding the adaptability of solutions to specific contexts.

Addressing environmental issues requires comprehensive, cross-disciplinary approaches to foster a more sustainable society. Within this context, microalgae biotechnologies have emerged as promising solutions, particularly when integrated into a circular bioeconomy framework. Microalgae, with their autotrophic metabolism, require water and nutrients such as nitrogen (N), phosphorus (P), and potassium (K), as well as inorganic carbon (C), for growth. In wastewater containing organic matter, microalgae can form a symbiotic relationship with heterotrophic bacteria, deleting organic C and releasing inorganic C necessary for photosynthesis. In turn, microalgae produce dissolved oxygen through photosynthesis, supporting bacterial activity. Some microalgae species also exhibit heterotrophic metabolism, utilizing organic C directly, even without light. These organisms are capable of accumulating a variety of valuable compounds, including proteins, pigments, antioxidants, polysaccharides, and lipids, which can be used for diverse applications such as biofuels, biofertilizers, biostimulants, and human and animal nutrition.^4^

In 2019, global algal biomass production (including seaweeds and microalgae) reached 35.8 million tons across 54 countries, marking a 60-fold increase since 1950.^5^ The Asian continent accounted for 97.4% of production, followed by the Americas (1.4%), Europe (0.8%), Africa (0.4%), and Oceania (0.05%).^5^ In Brazil, 17 companies produce or market raw materials and products derived from microalgae.^6^

Despite these advancements, microalgae cultivation requires significant inputs of water and nutrients, which can contribute substantially to production costs. Moreover, using industrial fertilizers in cultivation raises sustainability concerns due to the energy demands and greenhouse gas (GHG) emissions associated with their production.^7^ For example, the Haber–Bosch process, widely used for ammonia production, is responsible for 1–2% of global energy consumption and 1.44% of global carbon dioxide (CO_2_) emissions.^8^ To address these challenges, alternative sources of water and nutrients, such as domestic and industrial wastewater, are essential for scaling up microalgae production. Additionally, conducting cultivation under natural environmental conditions, such as ambient temperature and solar radiation, further enhances the feasibility of integrating microalgae-based wastewater treatment with biomass production. This approach aligns wastewater treatment with the generation of renewable bioproducts, significantly reducing the environmental footprint of their production chain. As such, microalgae represent a powerful tool for addressing pressing environmental challenges, particularly in developing countries, where tailored solutions are crucial for ensuring practicality and effectiveness.

Research into microalgae dates back several decades; however, significant interest in their sustainability and wastewater treatment application has grown markedly since 2010.^9^ After that, several studies have demonstrated the surprising capacity of microalgae-based systems for treating different types of wastewater with complex compositions. Microalgae can degrade organic matter through symbiotic interactions with heterotrophic bacteria. In landfill leachates, they contribute to nutrient removal and help attenuate toxicity. Microalgae also exhibit significant potential for the removal of heavy metals via biosorption and bioaccumulation mechanisms. Additionally, their ability to utilize carbon dioxide from flue gases supports photosynthetic growth while contributing to carbon mitigation.^10–12^ These capabilities of microalgae must be explored and understood to provide practical insights into their implementation. In this context, this manuscript highlights the progress achieved over 15 years of research in microalgae biotechnology at the Federal University of Viçosa, Minas Gerais, Brazil. The research group has established a distinctive approach to applying microalgae biotechnology to wastewater treatment, which has garnered international recognition^13,14^ and contributed significantly to the field. The study addresses multiple dimensions of this research, including biomass production, wastewater treatment technologies, bioenergy generation, agricultural applications, and environmental assessments. By synthesizing these findings, the manuscript demonstrates the potential of microalgae-based technologies to mitigate environmental challenges while also outlining the main barriers and opportunities for their implementation, especially in developing countries working toward universal access to sanitation services.

## 2 Wastewater Treatment and Microalgae Cultivation

Several technologies can be employed to effectively manage and treat wastewater, including adsorption processes, coagulation/flocculation, membrane filtration, and activated sludge systems.^15–21^ However, there is a growing interest in cost-effective treatment solutions that utilize natural systems.^22^ Nature-based solutions in wastewater treatment focus on designing systems that mimic and leverage the functionality of natural ecosystems with minimal reliance on mechanical components. These systems integrate plants, soil, porous media, bacteria, and other natural elements and processes to remove pollutants from wastewater.^23^ For instance, microalgae-based systems, such as high-rate algal ponds (HRAP), have demonstrated significant potential.

Additionally, these technologies contribute to advancing the circular economy by enabling the reuse and recovery of valuable byproducts, creating opportunities for innovative business models.^15^ Table 1 summarizes the group’s research over the past 15 years, focusing on microalgae biotechnology for wastewater treatment and biomass production. The studies are organized chronologically, reflecting the progression of research in microalgae biotechnology as it gained global prominence. Concurrently, efforts were made to propose solutions tailored to local demands and challenges associated with wastewater treatment.

**Table 1:** Performance of Different Reactors and Cultivation Strategies Regarding Pollutant Removal from Wastewater and Biomass Productivity^a^

			Pollutant removal efficiency (%)				Biomass growth		
Reactor	Wastewater	Observation	N-NH_4_^+^	Ps	CODs	TOCs	^b^VSS (g m^–2^d^–1^)	Chlorophyll-*a*	Study
HRAP	Domestic	HRAP with different shading	71	14	26	52	11.4	1.5 mg L^–1^	^24^
		HRAP with different shading and post-UV disinfection	74	19	30	55	9.3	2.1 mg L^–1^	
HRAP	Domestic	With biomass recirculation	76.82	17.99	10.94	53.38	6.99	0.17 g m^–2^ d^–1^	^16^
		Without biomass recirculation	81.77	15.72	8.35	54.23	6.87	0.18 g m^–2^ d^–1^	
HRAP	Primary meat processing	-	17.7–59.3	55.5–98.8	7.2–52	79.4–4.6	9.8–23.3	0.06–0.17 g m^–2^ d^–1^	^25^
	Secondary meat processing	-	90.6–100	33.3–94.4	0–43.3	-	5.1–10.6	0.06–0.14 g m^–2^ d^–1^	
HS	Domestic	Cotton as a support material and HRAP with 99% CO_2_	79.0	25.0	27.0	45.0	6.10	0.70 g m^–2^	^26^
HS	Domestic	Cotton as a support material	84.0	21.0	33.0	58.0	6.79	0.68 g m^–2^	
HRAP	Domestic	HRAP with 99% CO_2_	69.0	27.0	46.0	53.0	6.27	0.64 g m^–2^	
PBR bubble column	Primary meat processing	with CO_2_	Up to 100	Up to 100	30.8–54.9	39.6–70.3	26.5–52.5	10.9 mg L^–1^	^27^
	Secondary meat processing	with CO_2_	Up to 100	Up to 100	0–50	2.3–29.8	10.5–12.1	2.3–3.5 mg L^–1^	
BR	Domestic	Nylon as a support material	>90	70–76	-	-	1.80	0.02 g m^–2^ d^–1^	^28^
	Domestic	Polyester as a support material	>90	70–76	-	-	1.57	0.03 g m^–2^ d^–1^	
	Domestic	Cotton as a support material	>90	70–76	-	-	1.91	0.02 g m^–2^ d^–1^	
HRAP	Domestic	HRAP with 99% CO_2_	65.1	0	31.7	23.5	6.0	2.37 mg L^–1^	^29^
		HRAP with gasoline combustion gas	65.7	0	30.8	24.6	6.12	2.42 mg L^–1^	
HS	Domestic	Polyester as a support material	77.3	-	-	-	6.13	0.13 g m^–2^ d^–1^	^30^
HRAP	Domestic	-	75.3	-	-	-	3.68	0.05 g m^–2^ d^–1^	
HRAP	Cattle farming	-	99.8	53.2	55.7	83.9	7.12	1.64 mg L^–1^	^31^
HRAP	Domestic	HRAP with different depths (20, 30, and 40 cm)	47.7–84.4	7.7–48.3	40.4–42.2	62.8–64.2	2.12–4.3	0.04–0.05 g m^–2^ d^–1^	^32^
		HRAPs with different depths (20, 30, and 40 cm) and 99% CO_2_ addition	39.4–76.8	22–58.2	40.6–43.1	54.6–65.4	4.01–6.3	0.05–0.12 g m^–2^ d^–1^	
		40 cm HRAP with pre-UV disinfection	56.6	22.8	47.5	64.0	4.0	0.08 g m^–2^ d^–1^	
HRAP	Swine farming	Wastewater with different concentrations of Cu and Zn	66.1–92	36.4–82.8	-	-	-	25.42–50.53 mg m^–2^ d^–1^	^33^
HRAP	Blend (Domestic and paint booth)	Different blends of wastewater to improve C/N/P ratios	100	44.0–75.5	73.5–96.6	49.3–92.3	0.5–2.2	0.03 - 0.08 mg m^–2^ d^–1^	^34^
HRAP	Blend (Meat processing and brewery)	Different blends of wastewater to improve C/N/P ratios	41–99	-	-	68–78	4.3–5.3	-	^35^
HRAP	Swine farming	Wastewater with different concentrations of Cu	85–100	22–72	-	-	1.3–4.5	2.4–20.9 g m^–2^ d^–1^	^36^
HRAP	Swine farming	Wastewater with different concentrations of Zn	88–100	20.0–86.3	0	-	-	-	^37^
^c^HS	50% industry wastewater and 50% domestic wastewater	2-day scraping frequency	100	50.03	59.09		18.75	56.09 mg m^–2^ d^–1^	^38^
		4-day scraping frequency				92.95	9.28	31.34 mg m^–2^ d ^–1^	
		6-day scraping frequency					6.36	26.48 mg m^–2^ d ^–1^	
PBR	Industrial wastewater from juice processing and Municipal wastewater blending	C/N 30.67	100	65.5	79.8	80.2	21.4	0.08 g m^–2^ d^–1^	^39^
		C/N 20.82	100	71.1	74.1	78.8	23.6	0.07 g m^–2^ d^–1^	
		C/N 13.45	100	67.4	66.8	81.9	26.4	0.09 g m^–2^ d^–1^	
		C/N 7.52	76.3	68.8	88.9	75.6	40.0	0.21 g m^–2^ d^–1^	

a PBR = photobioreactor; HRAP = high-rate algal pond; BR = biofilm reactor; HS = hybrid system (HRAP + biofilm reactor); CO_2_ = carbon dioxide; C = carbon; N = nitrogen; Ps = soluble phosphorus; UV = ultraviolet; Cu = copper; Zn = zinc; N-NH_4_^+^ = ammonia nitrogen; CODs = soluble chemical oxygen demand; TOC = total soluble organic carbon; VS = volatile solids.

b For suspended and attached biomass, volatile suspended solids (VSS) and volatile total solids (VTS) were measured, respectively.

c Removal efficiency values refer to the hybrid system, while the algal growth values correspond to the biofilm biomass harvested at different harvesting frequencies.

Ammoniacal nitrogen (N-NH_4_^+^) and P compounds must be removed from wastewater to minimize their adverse effects on aquatic ecosystems, such as eutrophication. Organic matter, represented by chemical oxygen demand (COD) and total organic carbon (TOC), is also a critical parameter in wastewater treatment, as its presence contributes to the deterioration of water quality by reducing dissolved oxygen concentrations. In wastewater treatment, microalgae have emerged as efficient biological agents due to their ability to assimilate nutrients and organic compounds, which are incorporated into cellular biomass through well-defined metabolic pathways, including photosynthesis, bioaccumulation, biodegradation, biosorption, and enzymatic transformation.^40,41^ Table 1 summarizes the removal efficiencies of these parameters using different wastewater treatment strategies.

Inorganic forms of nitrogen (nitrate, ammonium) and phosphorus (phosphate) present in the effluent are absorbed by microalgae and converted into intracellular macromolecules such as proteins, lipids, and carbohydrates, resulting in the production of value-added algal biomass.^42,43^ The reductions in N-NH_4_^+^ are promising, ranging from 17.7% to 100%. The primary mechanisms driving this removal include assimilation by biomass, volatilization under suitable temperature and pH conditions, and nitrification. For P compounds, the reduction ranged from 0% to 100%. The absence of removal (0%) reflects cases where P content remained unchanged or increased. The primary removal pathways for P are assimilation and chemical precipitation. An increase in P concentrations in the culture medium can occur under conditions unfavorable for precipitation, such as variations in pH or insufficient saturation.

As is well-known, pH and temperature positively affect N removal, with studies in Table 1 showing a direct correlation between higher pH values and increased removal rates of free ammonia by volatilization. The concentration of dissolved oxygen in the medium can influence the nitrification process, as it is necessary for ammonia transformation into nitrate. For P, the pH effect is also described with higher removal rates via chemical precipitation, as high pH values were achieved during cultivation. However, the real conditions of microalgae cultivation in wastewater are dynamic, with multiple biological, chemical, and physical processes occurring simultaneously. This complexity makes it methodologically challenging to isolate and quantify the individual influence of specific variables on nutrient removal. Magalhães et al.,^44^ in a comprehensive review of experimental results on the performance of HRAP in nutrient removal, found that efficiencies vary widely and that identifying the dominant mechanisms can be complex.

Regarding COD and TOC, reported reductions ranged from 0% to 96.6% and 0% to 92.5%, respectively. Dissolved C in wastewater serves as an essential nutrient for microbial growth. In this context, microalgae-bacteria consortia are key in enhancing nutrient removal efficiency through mutual interactions and synergistic effects. Bacteria provide metabolites and inorganic C to support microalgae growth, while microalgae, through photosynthesis, supply oxygen needed for bacterial oxidation of organic matter. During the bioremediation process, microalgae fix carbon dioxide (CO_2_) and release oxygen (O_2_) under appropriate lighting conditions, thereby promoting medium oxygenation and enhancing the activity of aerobic bacteria responsible for degrading organic compounds.^42,45^ Systems that combine microalgae with heterotrophic bacteria have demonstrated significant synergies, leading to increased efficiency in the removal of nutrients and organic matter.^46^ This interaction fosters biomass production, ranging from 0.5 to 163.1 g m^–2^ d^–1^ in volatile suspended solids (VSS).

Pollutant removal efficiency and biomass productivity are strongly interconnected and influenced by the type of wastewater and cultivation reactors used, making it challenging to compare the results in Table 1 directly. Algal biomass productivity, for example, varies significantly due to differences in reactor configurations and measurement units used to evaluate algal growth. Nevertheless, the observed performance aligns with values reported in the literature for algal cultivation in domestic sewage (up to 100% N-NH_4_^+^, up to 100% P, and up to 81.0% COD)^44^ and swine effluent (>90% N-NH_4_^+^, 28–97% P, and 21–80% COD).^47^

### 2.1 Reactors

The group’s research primarily aims to explore low-cost alternatives with operational simplicity to address gaps in the literature and expand the boundaries of existing knowledge. In selecting the type of reactor to be used, various reactor designs were tested in the studies, focusing on overcoming key challenges such as achieving high productivity, facilitating easy biomass harvesting, and minimizing operational costs.^48^

Consequently, microalgae biomass production involved the use of HRAPs,^49–53^ photobioreactors,^54–57^ and hybrid cultivation systems.^58–60^

#### 2.1.1 High-Rate Algal Ponds (HRAPs)

HRAPs are open, shallow reactors known for their high biomass productivity, typically ranging from 10 to 30 g m^–2^ day^–1^. This performance is largely attributed to several key design and operational features. Continuous mixing by paddlewheels ensures uniform distribution of microalgae, optimizes exposure to solar radiation, and enhances gas exchange. Additionally, the shallow depth of the system maximizes light penetration throughout the water column, further promoting efficient photosynthetic activity and biomass growth.^61^ Due to their simplicity, ease of operation and maintenance, low implementation costs, and potential for utilizing produced biomass, HRAPs have emerged as a promising technology for universal sanitation. Their suitability is particularly notable in remote and hot climate regions, where land availability is generally not constrained, and sufficient solar radiation and temperatures favor algae growth. HRAPs provide decentralized, environmentally friendly, economically viable, and socially sustainable solutions. Drawing on studies in the literature,^49–53^ researchers have explored operational and engineering strategies to enhance wastewater treatment performance and biomass production, aiming to advance HRAP technology.

These studies utilized domestic and agro-industrial wastewater from economically significant activities in Brazil, particularly in Minas Gerais, where inadequate management poses a high pollution risk. In rural areas, especially on cattle farms, HRAPs have been used as low-cost, easy-to-operate systems to treat wastewater from stable cleaning while producing biomass.^62^ Using an influent containing 174 mg L^–1^ of total Kjeldahl nitrogen (TKN), 1144.1 mg L^–1^ of total phosphorus (TP), 729.9 mg L^–1^ of total suspended solids (TSS), and a surface application rate of 150 kg biochemical oxygen demand (BOD) ha^–1^ d^–1^, with a hydraulic retention time (HRT) of 12 days, the system achieved removal efficiencies of 99.8% for N-NH_4_^+^, 78.4% for TKN, and 53.2% for TP. The results suggested that wastewater polishing is necessary for organic matter before discharge into water bodies. The system’s productivity was approximately 7.12 g VSS m^–2^ d^–1^.

HRAPs have also been applied to treat wastewater from the meat processing industry, with two types of wastewater tested: primary postflotation and secondary postactivated sludge.^25^ Nutritional conditions influenced biomass production, with productivities of 23.3 g VSS m^–2^ d^–1^ and 0.175 g chlorophyll-a m^–2^ d^–1^ for primary wastewater and 10.6 g VSS m^–2^ d^–1^ and 0.14 g chlorophyll-a m^–2^ d^–1^ for secondary wastewater. HRAPs achieved efficient nutrient removal as secondary and tertiary treatments: 33% of N-NH_4_^+^ and 70.4% of TP in primary wastewater and 98% of N-NH_4_^+^ and 62% of TP in secondary wastewater. Similarly, Gama et al.^35^ evaluated primary flotation wastewater in HRAPs, observing 28% TOC removal, 50% N removal, and biomass productivity of 4.9 g VSS m^–2^ d^–1^. However, the high VSS concentration in this type of raw wastewater complicates its use as an indicator of biomass growth, as it affects the assessment of microalgae biomass health, as Veloso et al.^63^ noted. Organic nitrogen content decreased over time while chlorophyll-a concentrations increased.

Studies have also evaluated HRAP performance in treating wastewater from swine farming, which presents challenges due to high concentrations of N-NH_4_^+^ (2.8 g L^–1^) and suspended solids (13.3 g L^–1^).^64,65^ This wastewater is also known for its copper (Cu) and zinc (Zn) content (0.8–59.4 mg Cu L^–1^ and 0.5–234.1 mg Zn L^–1^)^47,66^ though these characteristics have been less studied. To address this, Oliveira et al.^33,36,37^ evaluated the removal of Cu and Zn in swine wastewater treated in HRAPs and investigated their effects on nutrient removal (N-NH_4_^+^ and TP). Results showed Cu removal efficiencies of 46–91%, primarily through precipitation, and Zn removal efficiencies of 78–99%, with biomass adsorption as a key mechanism. However, Cu and Zn compromised N-NH_4_^+^ removal, while TP reduction improved with adjustments to these metal concentrations. These findings highlight the distinct impacts of Cu and Zn on nutrient removal, emphasizing the need for further research into the effects of other metals commonly found in wastewater. This information provides a better understanding of the biochemical reactions occurring in HRAPs and informs the development of treatment strategies to improve HRAP performance. Such advancements are essential for achieving efficient and safe pollutant removal and ensuring HRAP systems are both effective and sustainable.

#### 2.1.2 Bubble Column Photobioreactor (PBR)

In addition to HRAPs, the bubble column photobioreactor (PBR) has been investigated to broaden the scope of opportunities within microalgae biotechnology. In this type of reactor, mixing is achieved by introducing gases, such as compressed air or CO_2_, at the base of the column, as shown in Figure 1. This process is critical for enhancing CO_2_ utilization efficiency by microalgae. Once introduced into the culture medium, the gases convert into bioavailable carbon forms, primarily carbon dioxide and bicarbonate, which are absorbed by microalgal cells via passive diffusion or active transport. It is important to note that the gas composition at the PBR inlet significantly influences both CO_2_ transfer rates and the pH of the medium. These parameters must be carefully regulated based on the gas source (e.g., ambient air, industrial CO_2_, or pure CO_2_) and the nature of the culture medium (e.g., wastewater or synthetic media).^67^ Furthermore, the use of transparent tubing in the design of this PBR technology allows uniform exposure of the culture medium to diffuse solar radiation, thereby enhancing the photosynthetic efficiency of the microalgae.^68^ Thus, due to the greater control and optimization of operational parameters in PBRs, such as gas introduction and concentration at the inlet and exposure to solar radiation, higher biomass productivities and microalgae densities can be achieved compared to HRAPs.

**Figure 1:**
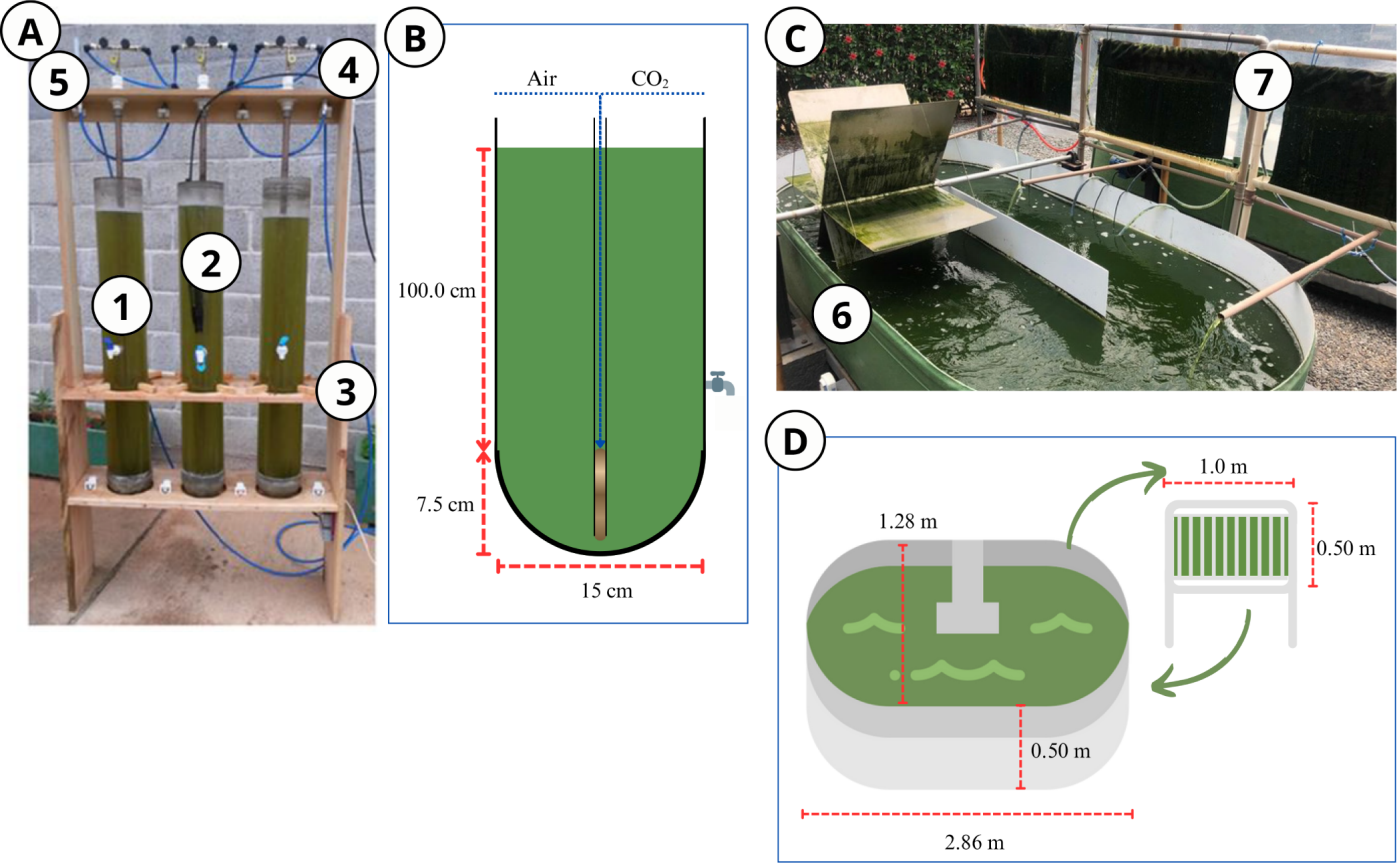
(A) Bubble Column Photobioreactor: (1) acrylic tube; (2) pH sensor; (3) wooden support structure; (4 and 5) flow meters. (B) Disperser and dimensions of each tube. (C) Hybrid System (HS) with suspended biomass growth in a high-rate algal pond (HRAP) (6) and adhered to a biofilm reactor (BR) (7) and (D) HS dimensions. Reproduced with permission from [Assemany et al., 2016; 10.1016/j.algal.2016.04.018].Copyright [2025] [Elsevier].

Tango et al.^27^ evaluated using bubble column PBRs with wastewater from the meat processing industry as a cultivation medium (Figure 1A,B). Productivity rates reached 52.5 g m^–2^ d^–1^ using wastewater from the flotation unit and 12.2 g m^–2^ d^–1^ using wastewater from activated sludge reactors. These differences were primarily attributed to the higher concentrations of nitrogen, phosphorus, and dissolved organic C in the primary wastewater.

From an environmental sustainability perspective, a comparison with HRAPs revealed significant drawbacks of bubble column PBRs, particularly related to their energy consumption and CO_2_ demand. These factors accounted for over 75% of the total impacts across the evaluated environmental categories.^69^

#### 2.1.3 Hybrid System

Biofilm reactors (BRs) have been widely used to enhance wastewater treatment and biomass production alternatives. These reactors enable greater utilization of atmospheric CO_2_ by facilitating direct contact between the biomass and the air^26^ and simplify biomass harvesting^19^ as the biomass grows attached to a surface through which the cultivation medium percolates continuously. In this context, a hybrid system (HS) was proposed, combining biomass adhered to a biofilm reactor (BR) and suspended growth in an HRAP, with the cultivation medium recirculated (Figure 1C,D).

In the study by Assis et al.,^26^ the initial BR configurations, including the choice of cotton as the support material, were based on the literature^70^ to replicate a reference BR under Brazilian experimental conditions (pilot scale, continuous flow, real domestic wastewater) and climatic conditions (exposed to environmental subtropical conditions). Two pilot-scale HSs were tested, one without CO_2_ supplementation and one with CO_2_ supplementation, and their performance was compared to a conventional HRAP with CO_2_ supplementation. All units used pretreated domestic wastewater from a full-scale up-flow anaerobic sludge blanket (UASB) reactor as the cultivation medium. The HS without CO_2_ supplementation achieved the highest total biomass production, 101.31 g m^–2^ (∼6.79 g m^–2^ d^–1^), while the HS with CO_2_ supplementation and the HRAP produced 72.86 g m^–2^ and 31.37 g m^–2^, respectively. The study concluded that the conventional HRAP with CO_2_ supplementation could be replaced by an HS with a BR, eliminating the need for CO_2_ supplementation. This result was attributed to the BR’s direct exposure to atmospheric air and solar radiation, which fulfilled the C demand of the algal biomass. Integrating a BR into a conventional HRAP offered operational simplicity and facilitated biomass harvesting through manual scraping.

However, Assis et al.^26^ identified several technical limitations (unpublished data), such as the durability of the cotton support material and the BR’s inclination, underscoring the need for further optimization. Subsequent studies^17,28^ explored these challenges at a reduced scale, using pretreated domestic wastewater from a UASB reactor.

L. R. de Assis et al.^28^ investigated the performance of different materials for supporting adhered algal biomass growth. The choice of support material significantly impacts the overall system’s performance.^18^ The authors evaluated support materials commonly cited in the literature for their ability to promote biofilm formation and algal productivity^17,28^ and their local availability. Cotton, nylon, and polyester fabrics were tested, with polyester demonstrating the best performance in terms of productivity (1.57 g m^–2^ d^–1^) and durability. Polyester’s abrasion resistance improved significantly after exposure to domestic wastewater, increasing from 7000 to 46 563 friction cycles. Consequently, polyester was selected for subsequent research on adhered growth.

In a follow-up study, L. R. de Assis et al.^17^ assessed algal biomass growth in reactors with different inclinations (15°, 45°, and 90°) using polyester as the support material. The experiment, conducted under real environmental conditions (such as radiation, precipitation, and wind) from July to December, spanned Brazil’s winter (dry season) and spring (rainy season). The cultivation medium was domestic wastewater from a UASB reactor operating in a separate sewer system without stormwater contributions. During dry periods, all reactors showed similar biomass productivities (∼1.50 g m^–2^ d^–1^). However, productivities differed significantly during rainy periods, with 0.21, 0.26, and 0.70 g m^–2^ d^–1^ at inclinations of 15°, 45°, and 90°, respectively, due to rainwater flowing parallel to the biomass, minimizing losses.

These findings informed the pilot-scale study by Assis et al.,^30^ which used pretreated domestic wastewater from a septic tank as the cultivation medium. The BR inclination was adjusted seasonally: during the rainy period (summer), the BR was set at 90° to the horizontal plane, while during the dry period (autumn and winter), it was operated at 15°. Integrating a BR with an HRAP and a settling tank (ST) resulted in total biomass production of 93.07 g m^–2^ and productivity of 6.13 g m^–2^ d^–1^, compared to 3.68 g m^–2^ d^–1^ in the conventional system without a BR. This demonstrated that integrating a BR into a conventional suspended culture system significantly improved algal biomass production, making it a promising technology for large-scale implementation.

BR studies have also highlighted the role of microalgae biotechnologies in nutrient removal. N-NH_4_^+^ removal efficiencies exceeded 78%.^17,26,30^ Soluble and total P removal efficiencies in HSs ranged from 16% to 30%,^26,30^ while smaller-scale BRs not coupled to HRAPs achieved soluble P removal rates of 70–76% under pH levels above 10.^28^ COD removal efficiencies ranged from 27% to 46%^26^ and 57% to 59%.^30^ Additionally, BRs supported conditions favorable for nitrifying bacteria, as evidenced by increased N-NO_3_^–^ concentrations in HSs (29.9–42.0 mg L^–1^) compared to HRAPs (17.8 mg L^–1^)

From an environmental perspective, life cycle assessment (LCA) studies emphasize the substantial environmental impacts of algae-based biofuel production, particularly during the cultivation and harvesting stages.^71^ LCA is a standardized methodology used to quantify the potential environmental impacts across all stages of a product’s life cycle.^72,73^ It is structured into four main phases: (i) goal and scope definition, (ii) life cycle inventory analysis, (iii) life cycle impact assessment, and (iv) interpretation of results^74^. By applying LCA, it is possible to identify the most environmentally critical processes and resource inputs, thereby supporting informed decision-making and the development of strategies to enhance environmental sustainability.

Thus, as studies from this environmental perspective were still scarce and required greater attention, life cycle assessments (LCAs) were conducted to evaluate the performance of a biofilm reactor and a hybrid system. Magalhães et al.^75^ demonstrated that cotton, as a support material, significantly contributed to environmental impacts due to insecticides in its production chain, affecting categories like global warming, fossil depletion, and terrestrial acidification. Assis et al.^76^ compared the environmental impacts of reactors using nylon, cotton, and polyester. While energy consumption during reactor operation contributed to most impacts, cotton accounted for over 90% of the impacts in categories like land use and ecotoxicity, corroborating Magalhães et al.^75^ Nylon and polyester had similar impacts, not exceeding 26% across categories.

Regarding the energy consumption reported by Assis et al.,^76^ it is important to highlight that while energy is required to operate the BR coupled with the HRAP, other studies underscore the energy advantages of biofilm-based systems for algal cultivation. For instance, Morales et al.^77^ demonstrated that a system comprising a rotating BR coupled with a pond consumed 16.7% less energy than a standalone pond. Furthermore, in a study evaluating energy valorization through anaerobic digestion (AD), only the scenario involving a biofilm cultivation system (rotating reactor) showed energy benefits, achieving a net energy ratio of 0.7, compared to 1.1 for a tubular PBR and 1.7 for HRAP.^78^

Additionally, comparisons between HS and HRAP revealed that HS systems were environmentally more favorable, contributing to approximately 28% reductions across all assessed environmental categories. These results were attributed to HS systems’ higher biomass recovery efficiency. Similarly, Xu et al.^78^ reported that the higher biomass concentration achieved through the rotating BR resulted in a 29.8% reduction in energy consumption during the biomass processing and AD stages compared to scenarios utilizing a tubular PBR. In alternative scenarios designed to promote adhered growth (by increasing the number or surface area of BRs within the HS), environmental impacts were reduced by approximately 24–40%,^76^ making microalgae biomass production from wastewater a more environmentally sustainable approach.

Notably, the studies by Magalhães et al.^75^ and Assis et al.^76^ relied on primary data, such as biomass productivity, harvesting efficiency, support material characteristics, and wastewater treatment performance, obtained from outdoor experiments conducted under Brazilian climatic conditions. These experiments used real wastewater as the cultivation medium and pilot-scale reactors (HRAP = 1000 L; BR = 0.5 or 1.0 m^2^). In contrast, secondary data, such as energy generation, were modeled using processes in the Ecoinvent database, reflecting large-scale operations. Detailed scope and life cycle inventory data are in the respective referenced manuscripts.

In light of these findings, future research is encouraged to advance the field by utilizing primary data from larger-scale systems to represent real-world operational conditions better.

### 2.2 Operational Strategies in HRAPs

HRAPs are among the most extensively studied and evaluated systems for cultivating microalgae in wastewater treatment.^79–82^ Despite their numerous advantages, such as simultaneous nutrient removal and biomass growth, HRAPs face critical limitations that hinder their broader adoption, including low biomass productivity and significant land requirements. The low productivity of HRAPs remains a bottleneck that, despite extensive research, has not yet been fully resolved, requiring further investigation and innovation. For example, Velásquez-Orta^83^ identified ammonium (NH_4_^+^) and light (photosynthetically active radiation: PAR) as key parameters influencing productivity. Similarly, Nordio et al.^84^ emphasized the effects of operational variables such as medium composition, pond depth, pH, and dissolved oxygen (DO) control, which impact the structure of microbial communities and nitrogen dynamics. Grivalský et al.,^85^ investigated strategies for PHB production, highlighting the importance of microbial population control and environmental conditions, such as UV disinfection and pH. Building on this body of research, the research group has focused on four main operational strategies to address the major bottlenecks in HRAP performance: (i) UV predisinfection of wastewater, which aims to suppress heterotrophic bacteria and pathogens that compete with microalgae or degrade system stability; (ii) Shading, to control excessive PAR and avoid photoinhibition under high irradiance conditions common in tropical regions; (iii) Depth adjustment, which affects light distribution, CO_2_ utilization, and the balance between land use and productivity; (iv) Optimization of the carbon/nitrogen/phosphorus (C/N/P) ratio, a critical factor for balanced growth and nutrient assimilation, particularly in carbon-limited domestic wastewater.

Each of these strategies is rooted in ecological and biochemical principles and has been evaluated in laboratory and pilot-scale HRAPs, as described in the following subsections. This section presents a critical synthesis of our findings and the literature, highlighting how manipulating operational parameters can optimize both biomass yield and pollutant removal, while maintaining system sustainability and scalability.

#### 2.2.1 Ultraviolet (UV) Predisinfection of Wastewater

In sanitary engineering, disinfection typically occurs at the end of the treatment process to ensure wastewater has reduced concentrations of solids and organic matter. However, applying UV disinfection prior to HRAPs has been proposed to lower the bacterial load entering the pond. This approach minimizes competition for space and nutrients between bacteria and microalgae, creating favorable conditions for algal growth and enhancing biomass production in HRAPs. The concept was first introduced by.^86^

Santiago et al.^24^ investigated HRAPs that received domestic wastewater treated by a UASB reactor, followed by UV disinfection at a 5.64 Wh m^–3^ dose. They compared the performance of HRAPs with and without UV pretreatment. The treatment efficiency for TOC (52–55%), COD (69–73%), N-NH_4_^+^ (71–74%), and soluble phosphorus (14–19%) was statistically equivalent between the two systems. However, the HRAP with UV pretreatment exhibited higher dissolved oxygen (DO) levels, increased pH values, and a greater percentage of chlorophyll-a in the biomass (0.95 ± 0.65% for the HRAP without UV vs 1.58 ± 0.65% for the HRAP with UV), indicating enhanced algal productivity. In contrast, the total biomass production, measured as VSS, was higher in the HRAP without UV predisinfection (11.4 g VSS m^–2^ d^–1^) compared to the HRAP with predisinfection (9.3 g VSS m^–2^ d^–1^).

Complementing this, Couto et al.^32^ explored the potential of UV predisinfection to allow for increased operational depths in HRAPs. Their study demonstrated that UV disinfection of wastewater in an HRAP operated at a depth of 40 cm resulted in algal biomass growth, organic matter removal (∼40%), ammonia removal (∼60%), and soluble phosphorus removal (∼20%) similar to those observed in a shallower HRAP (30 cm depth) without UV predisinfection. Additionally, the UV + HRAP system achieved a 4-log reduction in *Escherichia coli* (2 log units in each reactor), while the HRAP without UV pretreatment achieved only a 2-log reduction. The authors concluded that UV predisinfection could be a viable strategy to reduce land area requirements and enhance pathogen removal, enabling deeper HRAPs without compromising treatment efficiency.

However, an environmental assessment comparing UV predisinfection with other productivity-enhancement strategies, such as biofilm reactors and CO_2_ addition, revealed a significant contribution of UV lamps to the human toxicity category, primarily due to the presence of mercury in their production.^75^ Despite its technical viability, the UV predisinfection strategy requires further improvement, particularly in reducing the negative environmental impacts associated with the technology’s life cycle. This finding highlights the importance of conducting comprehensive environmental assessments when selecting strategies to enhance productivity in microalgae cultivation systems.

#### 2.2.2 Shading

Light availability is critical for the autotrophic growth of microalgae, as only PAR can be effectively utilized. Evaluating strategies to attenuate excessive radiation in HRAPs under natural tropical conditions is essential, as high PAR levels during certain months can hinder biomass growth due to photoinhibition. Shading also has practical implications for construction and operation, making it an important factor in optimizing system performance.

Santiago^87^ investigated the effects of shading on HRAPs by blocking 9%, 18%, 30%, 60%, and 80% of incident sunlight using high-density polyethylene meshes similar to those used in greenhouse construction. These shading levels corresponded to effective PAR intensities of 78%, 72%, 62%, 35%, and 22%, respectively. The meshes were supported by an external structure positioned 0.3 m from the pond’s edge. During the experiment, observed PAR ranged from 100 to 400 W m^–2^. The results revealed that intermediate PAR levels (achieved with 18% and 30% shading) supported higher photosynthetic activity, as extreme light intensities led to photoinhibition. These intermediate shading treatments resulted in the highest chlorophyll-a concentrations, though they were associated with lower total solids and VSS. This effect was likely due to ecological interactions between algae and bacteria in the ponds. Bacteria, which do not rely on light for growth, likely contributed to solid production, compensating for the reduced growth of photoinhibited microalgae.

Regarding wastewater treatment performance, the HRAP with 18% shading achieved the highest COD removal. However, COD removal did not exhibit a linear relationship with PAR levels, indicating that factors such as pH, temperature, and DO (although correlated with PAR) were not limited to organic matter degradation. Regarding *Escherichia coli* removal, the HRAP with 80% shading performed best, followed by the 30% shading treatment. This outcome suggests that moderate shading allowed deeper PAR penetration, enhancing the photoinactivation of pathogens through interactions between light waves, DO, pH, and photosensitizers.

In a related study, Couto et al.^88^ evaluated the effects of solar radiation intensity (blocking 9%, 18%, 30%, and 60%) on N assimilation by biomass in HRAPs. Their findings showed that nitrification was the primary N transformation process across all shading levels. Organic N removal was statistically similar between the control and the HRAP with 60% shading, despite a reduction in chlorophyll-a concentration under higher shading levels. This result highlights the role of heterotrophic bacteria in nitrogen assimilation, as they continue to function effectively even under lower light conditions. Thus, bacterial activity ensured stable N removal, compensating for reduced algal growth. However, PAR intensity influenced biomass composition, producing higher microalgae concentrations under lower shading levels.

These findings emphasize that intermediate PAR levels (achieved with 18–30% shading) optimize photosynthetic activity and treatment efficiency, while extreme shading (80%) enhances pathogen removal. These results underscore the importance of tailoring HRAP design and operation to local radiation conditions and ecological interactions to balance biomass production and wastewater treatment objectives. Furthermore, this research advances reactor configurations and designs by aligning with innovative approaches, such as dynamic and automatic light distribution systems for microalgae cultures.^89^ It also supports the development of low-cost photobioreactor materials with high transmittance and optimized refractive indices for maximum PAR refraction while reflecting long-wave thermal radiation.^90^

#### 2.2.3 Different Depths

Depth is a critical design and operational parameter in HRAPs, as it significantly affects radiation availability within the pond. As depth increases, the proportion of the water column exposed to light decreases for a given radiation intensity. Conversely, in HRAPs with shallower depths, the entire water column may be exposed to radiation, increasing the risk of photooxidation and photoinhibition. For large-scale HRAP applications, adjusting the depth can be a simpler alternative to shading screens, which require coverage over a larger surface area. Furthermore, optimizing depth is essential due to its direct influence on the surface area requirements of HRAPs.

Couto et al.^91^ evaluated CO_2_ transfer efficiency in HRAPs equipped with carbonation columns at 20, 30, and 40 cm depths. All HRAPs were operated in batch mode with the same surface area, resulting in different operational volumes. Depth is directly related to the surface area of the HRAP, and at reduced depths, the demand for the surface area can be very high. Therefore, depths of less than 20 cm were not tested. The study used domestic wastewater that had undergone anaerobic treatment. The results showed that depth did not affect CO_2_ dissolution in the carbonation columns or CO_2_ losses caused by paddlewheel movement. However, depth did influence CO_2_ assimilation efficiency. The HRAP with a depth of 20 cm demonstrated higher microalgae growth due to a greater proportion of the water column being exposed to sunlight, which increased CO_2_ assimilation.

In a subsequent study by Couto et al.,^32^ HRAPs with depths of 20, 30, and 40 cm were evaluated with and without CO_2_ supplementation. Shallower HRAPs (20 cm) exhibited higher N-NH_4_^+^ removal efficiencies (76% without CO_2_ and 84% with supplementation) and soluble P removal efficiencies (58% without CO_2_ and 48.3% with supplementation). Depth variations did not significantly affect organic matter removal (∼40% efficiency in all HRAPs), the dominant microalgae genera (*Chlorella* and *Scenedesmus*), or *Escherichia coli* inactivation (∼2 log unit removal). The 20 cm HRAP achieved chlorophyll-a production of 5.8 mg L^–1^ with CO_2_ supplementation and 4.3 mg L^–1^ without supplementation. In the 20 cm HRAP without CO_2_ addition, nutrient removal was primarily attributed to ammonia (N-NH_3_) volatilization and phosphorus precipitation. The study concluded that CO_2_ supplementation enabled greater depths in HRAPs, with algal biomass production at 40 cm depth comparable to that at 30 cm, without compromising wastewater treatment efficiency.

#### 2.2.4 Optimization of the C/N/P Ratio

Microalgal growth and nutrient removal are strongly influenced by the carbon/nitrogen/phosphorus (C/N/P) ratio, which depends on the availability of both organic and inorganic C sources. According to the literature, the optimal C/N/P ratio (by mass) for microalgal cultivation is 100:18:1.^92^ In contrast, domestic wastewater typically exhibits a C/N/P ratio of approximately 20:8:1,^44^ characterized by a relative deficiency in inorganic C compared to nitrogen. This imbalance can limit microalgal growth and reduce nutrient removal efficiency.

Strategies such as CO_2_ supplementation and wastewater blending have been explored to optimize the C/N/P ratio.^92^ CO_2_ supplementation provides an inorganic C source for microalgae, reduces the pH of the cultivation medium, and prevents nutrient loss, thereby enhancing cultivation performance. Couto et al.^32^ investigated the addition of CO_2_ in HRAPs treating domestic wastewater at depths of 20, 30, and 40 cm. Using carbonation columns with a height of 2.20 m, which prolonged gas–liquid contact time and improved CO_2_ dissolution efficiency, the study demonstrated increased chlorophyll-a production at all depths, likely due to the alleviation of C limitation for photosynthesis.

Despite its potential to mitigate C limitation, adding pure synthetic CO_2_ is economically and environmentally unsustainable on a large scale. Assis et al.^29^ evaluated alternative CO_2_ sources aligned with the circular bioeconomy framework to address this. They compared synthetic CO_2_ and gasoline combustion emissions delivered via carbonation columns and controlled the pH between 7.0 and 7.5. The results showed no significant difference in wastewater treatment efficiency or biomass composition between the two CO_2_ sources, indicating that additional gases in the emissions did not impair biomass productivity. However, an investment analysis revealed that synthetic CO_2_ or gasoline combustion gas was not economically viable for large-scale operations due to the negative net present value. The study highlighted the importance of scaling up and increasing biomass productivity to enhance process feasibility.

As noted by the authors, a key limitation of the research was the use of primary domestic septic tank effluents with high organic matter concentrations. These findings suggest that CO_2_ supplementation for microalgae cultivation in wastewater with a low C/N/P ratio should be further evaluated, considering alternative C sources and environmental and economic viability.

Blending different effluents has emerged as an effective strategy for C supplementation to improve the nutritional balance for algal development.^19,34^ This approach provides an additional C source and balances other nutrients necessary for algal growth while diluting toxic or inhibitory substances that could negatively impact algal metabolism.^19,34^

In this context, the blending of domestic wastewater with paint booth wastewater was evaluated in HRAPs. Domestic wastewater typically contains higher concentrations of nutrients such as nitrogen and phosphorus (106.7 mg L^–1^ of TKN and 6.8 mg L^–1^ of P) but lower total COD (588 mg L^–1^) and dissolved C (122 mg L^–1^). In contrast, paint booth effluent has lower TKN (3.1 mg L^–1^) and P (0.7 mg L^–1^) concentrations but higher COD (1,715 mg L^–1^) and dissolved C (326.1 mg L^–1^). Experimental optimization resulted in an ideal C/N/P ratio of 62:35:1, which achieved superior wastewater treatment outcomes, including 73% total organic C removal, 91% soluble COD removal, 100% N-NH_4_^+^ removal, and 76% soluble P removal, outperforming treatments using only domestic wastewater.^34^

Similarly, Gama et al.^35^ explored combining different wastewaters to optimize the C/N ratio in cultivation media. They blended meat processing wastewater with TOC, TKN, and P concentrations of 371.2 mg L^–1^, 69.2 mg L^–1^, and 10 mg L^–1^, respectively, with brewery industry wastewater, which contained 11 025 mg L^–1^ of TOC, 15.6 mg L^–1^ of TKN, and 102.4 mg L^–1^ of P. The study demonstrated that the removal efficiency of C, N, or P could be prioritized depending on the C/N ratio. For instance, C removal was maximized at a C/N ratio of 8.1, while N and P removal were most efficient at a ratio of 4.1. The brewery wastewater effectively increased C concentrations with minimal blending volumes. However, the authors emphasized the importance of controlling dilution rates to maintain treatment efficiency and avoid imbalances that could disrupt biological processes.

These findings underscore the potential of C supplementation through wastewater blending as a viable strategy for enhancing wastewater treatment using microalgae. Further research should focus on optimizing blending strategies, identifying sustainable C sources, and ensuring environmental and economic feasibility at scale.

#### 2.2.5 Separation and Harvesting of Biomass

Despite years of research demonstrating the potential of integrating microalgae production into wastewater treatment to address environmental issues, biomass separation and harvesting remain costly in the cultivation process.^19^ Consequently, the search for more efficient and cost-effective separation and harvesting technologies continues, particularly in the context of developing countries. Techniques based on physical mechanisms (e.g., sedimentation) and physicochemical processes (e.g., coagulation and flocculation) have shown promising applications in real wastewater treatment plants and have been tested for microalgal biomass separation.

However, it is important to emphasize that the choice of technique must be aligned with the main challenges associated with microalgae separation, including: the small cell size (<20 μm), the similarity between the density of microalgae and water, and their highly negatively charged surfaces (zeta potential).^93^ These characteristics result in low biomass concentrations (approximately 0.1–2 g dry weight L^–1^), with cells tending to remain suspended in the culture medium and exhibiting very low settling rates (<10^–6^ m s^–1^).^94^

In their study using untreated domestic wastewater, Li et al.^94^ found that hydrodynamic turbulence increased the autoflocculation efficiency of *Chlorella vulgaris* by 40–53.3% and enhanced the accumulation of extracellular polysaccharides (EPS), which act as bridging agents in the autoflocculation process. The maximum floc size and sedimentation velocity reached 373.5 ± 36.4 μm and 2.17 ± 0.29 m h^–1^, respectively. However, the study also revealed that autoflocculation efficiency is not directly proportional to the intensity of turbulence, as *Chlorella vulgaris* flocs began to disintegrate when exposed to shear stress above 0.0115 N m^–2^ and energy dissipation rates exceeding 1.25 × 10^3^ m^2^ s^–3^. Although the effects of hydrodynamics on microalgal flocculation remain somewhat uncertain, the authors achieved a biomass separation efficiency of approximately 94.5 ± 4.5% and proposed that understanding hydrodynamic control may represent a novel approach for cost-effective microalgal harvesting.

Microalgal cells possess negatively charged surfaces due to the presence of carboxyl and/or sulfate groups. As pH increases, the surface charge becomes more negative, which hinders flocculation.^95^ On the other hand, the autoflocculation of certain microalgae can be induced under alkaline conditions.^93^ Wu et al.^96^ demonstrated, using both freshwater and marine algae cultures, that the pH-induced harvesting mechanism involves the formation of magnesium hydroxide precipitates, which promote flocculation through charge neutralization and sweep flocculation. The precipitation of calcium and carbonate salts also contributes to this process.^96^ Additionally, autoflocculation can naturally occur in microalgal cultures exposed to sunlight and with limited CO_2_ supply,^97^ as these conditions enhance photosynthetic activity, which in turn increases the pH.

Within this context, our research group has evaluated various strategies for biomass separation and harvesting. These include sedimentation with biomass recirculation, sedimentation integrated with biofilm reactors, and coagulation/flocculation using both synthetic and natural coagulants. These approaches aim to improve biomass recovery efficiency while maintaining process feasibility for large-scale applications.

Costa et al.^16^ compared two HRAPs with a working volume of 1 m^3^, each followed by settlers (usable volume of 0.08 m^3^) under outdoor conditions. One system included biomass recirculation, while the other did not. Domestic wastewater from a UASB reactor, predisinfected with UV radiation and supplemented with CO_2_, served as the cultivation medium. The primary objective was to evaluate the impact of biomass recirculation on selecting dominant genera with improved sedimentation characteristics, ultimately enhancing biomass separation and system productivity. Over 3 months of monitoring, *Chlorella vulgaris* (56%) and *Scenedesmus acutus* (41%) were dominant in the HRAP with recirculation, whereas *Scenedesmus acutus* (90%) prevailed in the HRAP without recirculation. Despite these species’ poor sedimentation characteristics, sedimentation efficiency increased from 30.85% (HRAP without recirculation) to 46.89% (HRAP with recirculation).

A large-scale study in New Zealand similarly observed improved harvesting efficiencies with biomass recirculation. Domestic wastewater inoculated with *Pediastrum* sp. was treated in an HRAP (volume: 8 m^3^), followed by two settlers (ASC1 and ASC2) in series.^51^ Harvesting efficiencies exceeded 75% and 85% after ASC1 and ASC2, respectively, with recirculation, compared to 48% and 64% without recirculation. The superior performance was attributed to the dominance of *Pediastrum* sp. (annual average abundance > 90%), a species with excellent sedimentation properties, in the biomass recirculation approach. This dominance optimized harvesting efficiency (annual average > 85%) compared to systems without recirculation (∼60%).^51^

Costa et al.^98^ lower efficiencies compared to Park et al.^51^ are primarily attributed to differences in dominant species. These results likely influenced additional factors, including scale, settler design, climatic conditions, and monitoring duration. Ortiz et al.^20^ highlight that while experimental-scale studies on microalgal sedimentation are abundant, real-scale studies are scarce, making scaling-up sedimentation processes complex and emphasizing the need for further research in this area.

Castro et al.^16^ used sodium hydroxide (NaOH) as a coagulant to separate biomass from wastewater in the meat processing industry for use as fertilizer in millet cultivation. However, the NaOH requirement was significantly higher than traditional coagulants. Later, Souza et al.^21^ conducted a life cycle assessment and identified significant environmental impacts associated with NaOH use in the study by Castro et al.,^99^ highlighting the need for alternative harvesting methods.

Ferreira et al.^100^ conducted preliminary bench-scale tests to evaluate a tannin-based coagulant (15–20% concentration) due to its favorable results reported in the literature. This organic coagulant minimized issues related to biomass contamination, cell damage, and pigment alteration while preserving methane (CH_4_) production potential^101–104^ . Coagulation efficiency was assessed under varying pH levels (5–9), velocity gradients (86–200 s^–1^), and coagulant doses (0–100 mg L^–1^). The coagulant dose significantly impacted turbidity and COD removal (*p* < 0.05), with optimal results at 50 mg L^–1^ and pH 7, achieving 98% turbidity removal, 99% total suspended solids removal, and 92% COD removal.

As mentioned in Section 2.2, the operation of the HS optimized algal biomass production in a pilot-scale HRAP compared to a conventional HRAP, making it a promising technology for large-scale implementation, as highlighted by Assis et al.^30^ In addition to enhanced production, integrating a BR simplified the separation and harvesting of microalgae through manual scraping. The authors reported that suspended biomass yields in HRAPs operated alone and combined with a BR (forming the HS) were 2.64 g d^–1^ and 2.23 g d^–1^, respectively, statistically similar. In contrast, the BR alone achieved a significantly higher yield of 11.45 g d^–1^.

For both cultivation systems, suspended biomass was harvested via gravitational settling, while adhered biomass from the BR was manually scraped. Consequently, the biomass recovery from the HS reached 13.68 g d^–1^, with harvesting efficiencies of 61% for the HS and 22% for the standalone HRAP. In this study, harvesting from the BR was performed manually, which may pose challenges for large-scale implementation due to the significant labor requirements. Therefore, adopting automated technologies should be considered despite the increased energy and financial demands. Economic feasibility studies and scale-up assessments of BR systems are recommended to better evaluate their economic and technological viability and address these challenges.

Building on these findings, Ferreira et al.^105^ advanced the study of harvesting techniques from technical and environmental perspectives by comparing the HS and HRAP systems. Suspended biomass from the HRAP was harvested using either gravitational settling (GS) or tannin coagulation (TC), while biofilm was harvested manually through scraping. The evaluated scenarios were as follows:

●Scenario 1: Suspended cultivation in the HRAP with harvesting via GS.

●Scenario 2: Suspended cultivation in the HRAP with harvesting via TC + GS.

●Scenario 3: Cultivation in the HS (BR + HRAP) with suspended biomass harvested via GS and attached biomass from the BR harvested through manual scraping.

The separation efficiencies for suspended biomass using GS were 19.84% and 21.24% for the standalone HRAP and the HS, respectively. In contrast, adding TC before GS increased separation efficiency to 99%, while biofilm harvesting via manual scraping was considered 100% efficient. Biomass recovery efficiencies of 87.44%, 98.99%, and 99.58% were achieved for GS, TC + GS, and GS + BR, respectively. From a technical standpoint, TC and BR significantly optimized the harvesting process.

From an environmental perspective, Scenarios 2 and 3 demonstrated lower environmental impacts across all categories evaluated using the ReCiPe methodology. In Scenario 1, the lower biomass concentration factor necessitated higher energy inputs to produce sufficient biomass for the harvest of 1 kg (the functional unit adopted). By contrast, the energy consumption in Scenarios 2 and 3 was approximately five times and 1.6 times lower than in Scenario 1, respectively, highlighting the advantages of incorporating TC and BR.

The authors also evaluated the scenarios’ scale-up potential and emphasized the importance of developing automated technologies for biofilm harvesting. This aspect requires further investigation from both technical and sustainability perspectives to identify optimal solutions for large-scale applications.

## 3 Characterization of Biomass Obtained from Cultivation in Wastewater

### 3.1 Phytoplankton Community

Among the numerous studies conducted by the research group, the genus *Chlorella* sp. was consistently detected, regardless of the season, type of wastewater, mode of operation (continuous or batch), or cultivation strategy (Figure 2).

**Figure 2:**
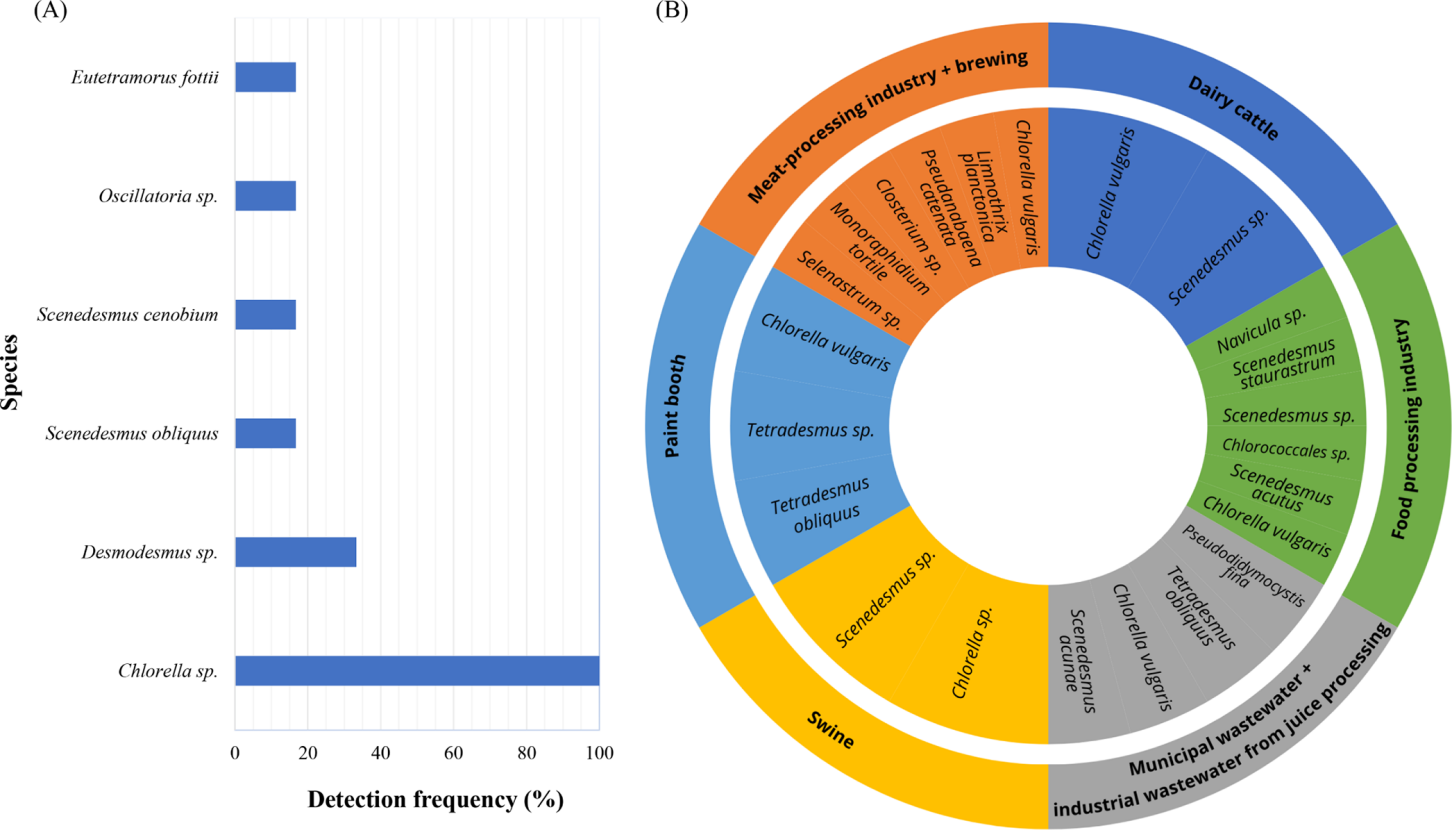
(A) Frequency of detection of phytoplankton genera and/or species with relative abundance exceeding 20% in studies utilizing HRAPs and domestic wastewater as the cultivation medium. (B) Phytoplankton genera and/or species have relative abundance exceeding 20% in various wastewater type studies. Data from studies employing domestic wastewater:^24,26,29,30,32,39,104,106–109^ studies that used other wastewaters:^25,27,31,33,35–37,39,109–113^ .

In an 11-month monitoring study, Santiago et al.^24^ reported a greater abundance of *Coelastrum* sp. and *Micractinium* sp. in June, with dominant genera (*Chlorella* sp. and *Desmodesmus* sp.) reappearing shortly thereafter. Similarly, Assemany et al.^108^ observed higher populations of *Desmodesmus* sp. during summer and autumn, while *Chlorella* sp. dominated in winter and spring. In colder months (May to August), *Eutetramorus fottii* exhibited a relative abundance of around 40% in the study by Assis et al.,^30^ while the genus *Oscillatoria* was prominent in March. *Scenedesmus obliquus* was detected by Couto et al.,^32^ with 90% relative abundance in free-floating cells within a 20 cm deep HRAP in March. In bioreactors (BRs), *Chlorella vulgaris* was the most abundant genus, with a relative abundance exceeding 60%.^26,30^

Oliveira et al.^37^ reported an exception to *Chlorella* dominance, who observed *Scenedesmus* sp. as the only genus present in HRAPs treating swine wastewater with varying Zn concentrations. Similarly, *Scenedesmus* sp. was the sole genus identified in bubble column PBR operations,^27^ where high densities of fungi and bacteria were also reported. *Navicula* sp. (a diatom genus) was also identified with 37.4% relative abundance in batch operations treating meat processing wastewater postprimary flotation.^113^

The findings underscore *Chlorella* sp.‘s remarkable presence and predominance, highlighting its adaptability and resilience. Regardless of seasonal variations, wastewater characteristics, operational strategies, or wastewater types, *Chlorella* sp. consistently emerged as the most frequent genus. Its versatility underscores its importance in treatment systems, offering effective nutrient removal while thriving in diverse environments.

Using indigenous species without prior inoculation for domestic wastewater promotes the selection of more tolerant and adaptable species, enhancing the robustness and stability of wastewater treatment over time. However, when biomass utilization extends beyond wastewater treatment, the lack of species selection through inoculation may negatively affect biomass characteristics for the intended final application. Therefore, integrating the biomass valorization strategy with its biochemical characterization is crucial. These characteristics are influenced by the phytoplankton community and the cultivation medium and operational conditions employed (see Section 2.2).

This approach differs from synthetic cultivation practices, where the biomass destination is predetermined, and cultivation is tailored entirely toward the valorization pathway. However, incorporating inoculation and maintaining a predominant species during HRAP operation may render the technology technically and economically unfeasible for wastewater treatment plants. Conversely, inoculation has been a standard practice in nondomestic wastewater treatments to ensure biomass growth and expedite culture startup in wastewater with higher organic matter and suspended solids concentrations than domestic wastewater.

Lastly, most identified phytoplankton genera exhibit mixotrophic metabolism. This suggests that, beyond the symbiotic relationship between microalgae and bacteria (with oxygen provided by microalgae and CO_2_ by bacteria), the phytoplankton community also contributes to organic matter degradation in wastewater, particularly during periods of low or limited light availability.

### 3.2 Biomass Biochemical Composition

The biochemical characterization of biomass from wastewater treatment is essential for defining its potential applications, such as producing biofuels, food, chemicals, and fertilizers. Key components analyzed include carbohydrates, lipids, proteins, and ash.

The geographical location and specific environmental conditions where microalgae are cultivated significantly influence their biochemical composition. In particular, most studies conducted in Viçosa, Minas Gerais, Brazil, highlight the critical role of the cultivation medium in shaping the biochemical makeup of algal cells. The initial characteristics of the wastewater directly impact nutrient availability for the algae, and variations in nutrients like N and P can alter algal cell composition.^114^ The research used various wastewater types, resulting in diverse cultivation media and distinct biochemical profiles (Figure S1).

The findings indicated that, regardless of the type of effluent or reactor configuration, the biomass exhibited an average protein content ranging from 32% to 42%, followed by carbohydrate content (18–23%) and lipid content (13–16%). Although the effluent type can influence the absolute levels of these macronutrients, the relative proportions generally remained consistent: protein content was higher than carbohydrate content, which in turn was higher than lipid content. This persistence in biochemical composition is largely attributed to the microalgal species found, which were similar across the studies analyzed.

A notable example is the use of agro-industrial effluents (including those from pig farming, slaughterhouses, and beef cattle operations), which resulted in the highest levels of proteins, carbohydrates, and lipids. These effluents typically contain higher concentrations of nitrogenous compounds,^33^ contributing to the elevated protein content in the algal biomass. Furthermore, biomass cultivated in agro-industrial effluents exhibited lower ash content compared to that derived from domestic wastewater, resulting in a higher proportion of organic material. This characteristic is especially favorable for biofuel production, as high ash content is known to impair fuel quality.

Regarding carbohydrates, the observed levels align with the recommended range for bovine feed supplementation (13–22%). However, lipid content was lower than typically reported for algal biomass targeted for energy applications^115^ (refer to Section 4.1.1 for further details). This can be attributed to the high nutrient availability in wastewater and competition for space, light, and nutrients among microalgae and other microorganisms, limiting lipid accumulation in the biomass.

Proteins represent one of the most valuable byproducts of wastewater-grown microalgae, particularly for human and animal nutrition. Madeira et al.^116^ emphasized that the protein from *Chlorella vulgaris*, a species commonly found in wastewater, exhibits quality comparable to that of yeast, soybean meal, and milk proteins. However, precautions regarding the sanitary safety of protein products derived from wastewater-grown biomass must be taken when intended for food applications.

No significant differences were observed when comparing the biochemical composition of suspended and adhered biomass in hybrid systems.^30^ Similar results were obtained when different support materials (nylon, polyester, and cotton) were used in biofilm.^28^ The biomass characteristics included higher protein content (23–32%) and ash (20–29%), followed by carbohydrates (11–22%) and neutral lipids (2.9–10.6%).^26,28,30^

Despite the operational strategies adopted in the compiled studies, the biochemical composition of biomass showed minimal variation, with wastewater chemical composition being the most significant influencing factor. Moreover, while there is extensive literature on lipid fraction valorization for biofuel production^117^, the potential for fully utilizing wastewater-grown biomass to obtain multiple valuable products, particularly protein-based byproducts, remains underexplored^118^. These findings underscore the need for further research to develop comprehensive biomass valorization routes on a larger scale.

## 4 Biomass Valorization

In parallel with investigating the performance of microalgae in wastewater treatment and the production and harvesting of algal biomass, the research group has also focused on evaluating the potential utilization routes for this biomass. This research aims to add value to treatment byproducts, enhancing sanitation services’ economic and environmental appeal. Adopting this approach aims to attract greater investment in the sector, establish more advantageous business models, and contribute to the universalization of these services, particularly in economically disadvantaged countries.

The recovery of resources from sanitation systems and the promotion of a circular economy through the development of bioproducts align with the ecological transition toward a society that is less dependent on fossil fuels. This underscores the importance of ongoing research in this field.

The proposed utilization routes primarily focus on bioenergy and agricultural applications, often considered less advanced uses of biomass. However, these applications are justified by the origin of biomass and the minimal process interference they require. For instance, the use of indigenous species and the simplicity of maintenance and operation of cultivation reactors within wastewater treatment plants make these routes practical and cost-effective.

### 4.1 Energy

The increasing demand to replace fossil fuels with alternative and renewable sources in the global energy matrix, driven by worsening climate and environmental challenges, has accelerated the development of energy valorization pathways for biomass derived from wastewater treatment. The research group has actively pursued this approach, aiming to remain at the forefront of innovation. Integrating wastewater treatment with bioenergy production through microalgae biotechnology presents a significant opportunity, particularly for developing countries. In addition to enabling resource recovery, this approach can reduce GHG emissions and dependence on fossil fuels. It also fosters the adoption of technologies tailored to specific contexts, helping these countries achieve technological autonomy and positioning them as leaders in the energy transition.

The energy crisis that emerged after World War II and the high cost of fossil fuels spurred research into using algal biomass for biodiesel production.^119^ However, the limited accumulation of neutral lipids, largely due to the nutrient availability commonly found in wastewater, led to the exploration of alternative pathways, particularly those leveraging wet biomass.

#### 4.1.1 Lipid Extraction

The research group has focused on lipid extraction for biodiesel production. The main challenges of this route are the low lipid content in biomass produced during wastewater treatment (Section 3.2) and the necessity of drying the biomass prior to energy conversion. Twelve HRAPs were operated in parallel to investigate the impact of varying light intensities and UV predisinfection on the lipid characterization of microalgae cultivated in HRAPs treating domestic wastewater to address these issues. Half of the HRAPs used untreated UASB effluent, while the other half employed UASB effluent that had undergone UV disinfection. Additionally, the units were covered with shading screens to block 9%, 18%, 30%, 60%, and 80% of solar radiation.^108,120,121^

In HRAPs with UV predisinfection, lipid productivity decreased when solar radiation was blocked by more than 30%. The units with no shading and those blocking 9% and 30% of solar radiation demonstrated statistically higher lipid productivity at 0.9 g m^–2^ d^–1^. UV predisinfection increased the presence of algae in the total biomass, and fatty acid unsaturation levels rose as solar radiation exposure decreased.^108^ When comparing HRAPs with and without UV predisinfection (under no solar radiation blocking), the UV system positively influenced lipid accumulation (9.5% versus 8.6%, respectively) and chlorophyll-a concentration (2.5 mg L^–1^ vs 1.7 mg L^–1^). However, by limiting the concentration of other microorganisms in the pond, the UV-treated units showed lower VSS concentrations, resulting in similar lipid productivity across both setups.^121^

Lipid accumulation under both conditions (with and without UV disinfection) was influenced by the concentrations of VSS, chlorophyll-*a*, and solar radiation.^120^ HRAPs without UV disinfection achieved better results in total biomass production and lipid accumulation.^120^ C16, C18:1, and C18:3 fatty acids dominated the lipid profile across all conditions. The oils produced in the HRAP with no UV disinfection and 60% solar radiation blocking, as well as those from the HRAP with UV disinfection and no solar blocking, were most suitable for biodiesel production due to their favorable characteristics, including oxidative stability, freezing point, and lower percentages of polyunsaturated fatty acids.^120^ The total lipid content did not vary significantly among the HRAPs, averaging 9.5%. Consequently, strategies to enhance biomass productivity, rather than lipid accumulation, are identified as the most effective approach for biodiesel valorization.^108,120^ Additionally, other strategies, such as wastewater blending or CO_2_ supplementation (Sections 2.2.4 and 2.2.5), could effectively increase lipid content in the biomass and address the C limitations in some wastewater, especially when combined with nitrogen starvation.

#### 4.1.2 Briquettes

Due to the low lipid content of microalgae derived from effluent treatment, the group explored alternative energy conversion routes, testing the use of biomass for direct combustion in briquettes. In this context, Costa et al.^122^ produced briquettes by combining the epicarp of *Jatropha curcas* L. (physic nut) with microalgae biomass obtained from agro-industrial wastewater treatment. Including microalgae biomass improved the energy density of the briquettes, with the optimal composition being 50% microalgae biomass sourced from meat processing industry wastewater. This specific biomass demonstrated superior calorific value (20.4 MJ kg^–1^), volatile matter content (69%), density (623.1 kg m^–3^), energy density (10 512 MJ m^–3^), and reduced ash content (22.8%) compared to briquettes made from biomass derived from HRAPs and BRs cultivated in domestic wastewater. However, to meet the minimum requirements for briquette production, the biomass had to be dried to a moisture content of 12%.

The implications of the biomass drying process for energy balance and potential environmental impacts were further analyzed by Marangon et al.^123^ The drying stage accounted for 99% of emissions associated with climate change, freshwater eutrophication, and fossil resource depletion. This intensive energy requirement resulted in a negative energy balance, highlighting the critical need to investigate alternative drying methods with lower energy demands. Based on their findings, the researchers suggested exploring other processing routes that minimize the drying requirement, such as AD and hydrothermal processes, including liquefaction and carbonization.

#### 4.1.3 Anaerobic Digestion

Anaerobic digestion is a well-established biochemical process for converting organic waste into biogas. It performs particularly well in tropical climates but is employed in temperate and subtropical regions. In these latter contexts, heating the digester is often necessary to enhance process efficiency, although it requires additional energy. AD is well-suited for processing wet substrates, making it highly applicable for treating agro-industrial waste, particularly in isolated and rural areas of developing countries.^124^ Additionally, this method has been explored as an alternative for valorizing dry biomass, previously investigated through lipid extraction and briquette production experiments. Considering these advantages and the region’s tropical climate, the research group has been actively developing studies involving AD.

Assemany et al.^107^ evaluated thermal and lipid extraction pretreatments and substrate complementarity as strategies to enhance biogas yield. Lipid extraction significantly increased biogas production (enfold) by making intracellular content more accessible. However, energy analysis of an integrated route combining lipid extraction and AD revealed that lipid extraction contributed only to marginal energy gain, primarily due to the low lipid accumulation in biomass produced in wastewater systems. As a result, lipid utilization was deemed energetically infeasible, with biogas production from crude biomass emerging as the most favorable energy route. This was true for biomass cultivated in HRAPs using domestic wastewater^107^ and biomass grown in a bubble column PBR using wastewater from the meat processing industry.^125^

The complementarity of substrates was also evaluated by codigesting olive mill wastewater with algae biomass produced in HRAPs. The HRAP biomass was cultivated using the brewery industry and domestic wastewater as culture media. In batch tests, biomass from domestic wastewater achieved a methane yield of 0.10 m^3^ CH_4_ kg^–1^ volatile solids (VS), with a 61% increase in CH_4_ production when codigested with 10% (v/v) olive mill wastewater compared to monodigestion. The addition of olive mill wastewater mitigated the negative effects of the high ash (40%) and low carbohydrate (3.6%) content in the wastewater-grown biomass during AD.^126^ Conversely, monodigestion of biomass cultivated in brewery wastewater resulted in a higher CH_4_ yield (0.16 m^3^ CH_4_ kg^–1^ VS), 14% greater than codigestion with olive mill wastewater. This outcome was attributed to the more balanced chemical composition of the brewery-cultivated biomass, underscoring the importance of assessing the chemical constitution of algal biomass, which is largely influenced by the cultivation medium, when planning AD processes^126^

On a larger scale, biogas production and organic matter degradation efficiency were assessed over three phases (72 days) in an anaerobic hybrid reactor (2.8 L). This study involved using olive mill wastewater as a complementary substrate to algal biomass and thermal pretreatment to disrupt algal cell walls. Methane yield during monodigestion of algal biomass was 0.08 L CH_4_ g^–1^ VS, compared to 0.25 L CH_4_ g^–1^ VS for codigestion with 10% (v/v) olive mill wastewater and 0.21 L CH_4_ g^–1^ VS for thermal pretreatment followed by codigestion.^127^ Flow cytometry analysis revealed that the thermal pretreatment disrupted only 37.2% of the cell walls, and the short exposure time (10 min) was insufficient to improve methane yields significantly. Under the evaluated conditions, substrate complementarity yielded better results than thermal pretreatment.

The anaerobic hybrid reactor performed strongly in removing organic matter when olive mill wastewater was added (58–66%).^127^ However, reactor performance was compromised when digesting algal biomass alone, mainly due to the loss of active anaerobic biomass and the high particulate fraction of the substrate, which was unsuitable for this reactor type. Additionally, the low biodegradability of algal biomass remains a critical challenge to overcome.^127^

#### 4.1.4 Hydrothermal Processes

The study of hydrothermal liquefaction (HTL) within the context of microalgae biotechnologies is primarily justified due to its ability to convert organic matter into bio-oil under subcritical temperature and pressure conditions. This approach reduces the dependency on lipid or carbohydrate content in the biomass, as seen with AD. Additionally, HTL operates in the presence of water, eliminating the need for complete biomass drying, which represents an energy-efficient alternative to challenges faced in biodiesel production. The unique advantages of HTL have sparked significant interest within the research community, prompting the group to carry out and continue research in this area.

Couto et al.^128^ evaluated bio-oil production via HTL using biomass from domestic wastewater treatment in HRAP. They investigated various temperatures, reaction times, and biomass-to-water ratios. The study revealed that extending reaction time beyond 15 min or increasing temperature beyond 300 °C did not significantly improve bio-oil yield. Optimal conditions (300 °C, 15 min, and a biomass-to-water ratio of 1:10) yielded a bio-oil production of 44.4% (ash-free dry basis).

Further analysis by Couto et al.^106^ assessed the impact of different ash contents on bio-oil yield (see Figure S2). This evaluation was crucial given the high ash content often observed in biomass derived from domestic wastewater. The study found that biomass with lower ash content achieved higher bio-oil yields on a dry basis, with 34.7% and 25.3% yields for biomass containing 21.6% and 40% ash, respectively. However, the ash-free dry basis yield remained consistent at 45.3% and 44.4% for biomass, with 21.6% and 40% ash. This indicates that ash content did not interfere with the decomposition of volatile material during HTL but reduced bio-oil yield on a dry basis by decreasing the volatile material available for reaction. The ashes, primarily composed of silica, may not hinder the conversion process and could potentially act as HTL catalysts, depending on their composition. Future studies could explore the catalytic role of ash in HTL or strategies to remove ash before HTL to optimize bio-oil production.

Despite the demonstrated potential of producing bio-oil from wastewater-grown biomass, the final product has characteristics limiting its application as a fuel. For example, Couto et al.^128^ reported a minimum nitrogen recovery of 57% in the bio-oil, which could lead to elevated nitrogen oxide (NOx) emissions during combustion. Additionally, HTL generates solid residues, an aqueous phase (AP), and gases^7,129^ . In an LCA of biofuel production from HTL-derived bio-oil, the most impacted environmental category was marine eutrophication due to nitrogen compound emissions in the AP.^130^ Effective utilization of these byproducts is critical for the economic and environmental sustainability of HTL.

Solid residues from HTL consist of inorganic materials and undegraded organic compounds. Couto et al.^128^ reported that these residues exhibited an ash content ranging from 82.1% to 92.6%, with elemental compositions of C varying from 5.4% to 9.6%, O from 0.2% to 8.7%, H from 1.2% to 1.7%, and N from 0.3% to 0.7%. Another hydrothermal process, hydrothermal carbonization (HTC), offers a complementary pathway by converting wet biomass into hydrochar, a C-rich material. Hydrochar has various applications, including use as a biofuel, fertilizer, soil conditioner, or raw material for gasification and activated C production.^131^ Castro et al.^113^ studied HTC using biomass from meat-processing wastewater and identified that the optimal energy recovery (78.21%) and solid recovery (77.72%) were achieved at 170 °C for 10 min. However, lower temperatures (130 °C) with longer reaction times (50 min) produced hydrochar with higher nitrogen and phosphorus content, indicating potential as an agricultural biofertilizer. Subsequent LCA and economic analyses concluded that hydrochar as a biofertilizer was environmentally viable, whereas its use as a solid biofuel was economically unfeasible.^132^

Similarly, Oliveira et al.^133^ demonstrated that HTC at 170 °C for 10 min of microalgae biomass cultivated in swine wastewater achieved energy recovery rates of 49–63%. Acid washing pretreatment further increased energy recovery to 73.5% but doubled the nitrogen and sulfur content in hydrochar, raising concerns about greenhouse gas emissions during combustion. However, these same properties (nitrogen, sulfur, and phosphorus content) enhance hydrochar’s suitability as a soil amendment, corroborating the findings by Castro et al.^113^ regarding its agricultural potential.

Another consideration for HTC is the management of byproducts such as AP and gaseous emissions. The AP contains solubilized organic and inorganic compounds, including macronutrients C, N, and P^133^. Effective management of the AP is essential to prevent environmental harm, and it has potential applications as a medium for microalgae cultivation or substrate for AD. Marin-Batista et al.^134^ demonstrated that combining HTC with AD of the AP could achieve a net energy recovery of 91%.

The gaseous phase of HTC is primarily composed of CO_2_ and is often less emphasized in studies due to its minor contribution to mass balance. For example, Taufer et al.^135^ reported that after HTC of digestate, 72% of C remained in hydrochar, 21% transferred to the AP, and only 7% transferred to the gaseous phase. These findings highlight the need for holistic strategies to maximize the benefits of HTC while mitigating its drawbacks, paving the way for sustainable bioeconomy applications.

#### 4.1.5 Future Perspectives on Energy Valorization of Wastewater-Grown Algal Biomass

Table 2 presents the above-mentioned results, and the future prospects for each energy conversion route are discussed in detail.

**Table 2:** Energy Yields from Various Valorization Routes of Wastewater-Grown Microalgae Biomass over 15 Years of Research

Route	Wet or dry	Origin of biomass	Operating conditions	Yield	Reference
Lipid extraction	Dry	Domestic wastewater with ultraviolet (UV) disinfection	Solar radiation blocking	8.9–10% of lipid content/0.61–0.96 g lipid m^–2^ d^–1^	^108^
		Domestic wastewater with UV disinfection	-	7.6% of lipid content/0.2 g lipid m^–2^ d^–1^	^107^
Briquettes	Dry	Domestic wastewater	-	13.21–20.36 MJ kg^–1^	^122^
Anaerobic digestion	Wet	Domestic wastewater with UV disinfection	250 mL batch test of monodigestion of algal biomass	0.20 m^3^ CH_4_ kg VTS^–1^	^107^
		Domestic wastewater with UV disinfection	250 mL batch test of monodigestion of algal biomass after lipid extraction	2.6 m^3^ CH_4_ kg VTS^–1^	
		Domestic wastewater	71.5 mL batch tests of mono digestion of algal biomass and codigestion with olive mill wastewater	0.06–0.10 m^3^ CH_4_ kg VTS^–1^	^126^
		Brewing industry wastewater	71.5 mL batch tests of mono digestion of algal biomass and codigestion with olive mill wastewater	0.07–0.16 m^3^ CH_4_ kg VTS^–1^	
		Brewing industry wastewater	2.8 L semicontinuous tests of mono digestion and codigestion of algal biomass with 10% olive mill wastewater	0.08–0.25 m^3^ CH_4_ kg VTS^–1^	^127^
			2.8 L semicontinuous tests of heat-pretreated algal biomass with 10% olive mill wastewater	0.21 m^3^ CH_4_ kg VTS^–1^	
Hydrothermal liquefaction	Wet	Domestic wastewater	Reaction temperature: 275, 300, and 325 °C; reaction time: 15, 30, and 45 min; Biomass/water ratio: 1/20, 1/10, and 1/5 (w.w^–1^)	37.4–44.4% bio-oil (dry ash-free basis)/38.1 MJ kg^–1^	^128^
Hydrothermal carbonization	Wet	Meat processing industry wastewater	Reaction temperature: 130, 150 and 170 °C; reaction time: 10, 30, and 50min; Biomass/water ratio: 1/10 (w.w^–1^)	67–77% hydrochar/16.99–17.47 MJ kg^–1^	^113^
		Swine wastewater	Reaction at 170 °C for 10 min with biomass/water ratio of 1/10 (w.w^–1^)	60.1% hydrochar: 16.9 MJ kg^–1^	^133^
			Reaction at 170 °C for 10 min with biomass/water ratio of 1/10 (w.w^–1^), using biomass after acid washing to remove ash	71.2% hydrochar: 23.6 MJ kg^–1^	

Recently, lipid accumulation in microalgae has continued to be a topic of interest, with high lipid yields reported for biomass grown in wastewater. Tango et al.^27^ achieved a lipid content of 26.5% in *Scenedesmus obliquus* cultivated for 16 days in municipal wastewater. Zhao et al.^114^ reviewed various studies on biodiesel production from wastewater-grown microalgae, reporting lipid contents ranging from 17% to 38%.

Stress-inducing strategies, such as variations in cultivation conditions, chemical additives, and abiotic stressors (e.g., nutrient limitation, metal exposure, light intensity), are well-known methods for promoting lipid accumulation.^114^ One common approach is diluting wastewater to induce nutritional stress, which has been shown to enhance lipid content. For instance, this strategy resulted in lipid contents of 34.8% and 31.3% in biomass comprising *Chlorella* sp. and *Scenedesmus* sp.^33^ and a consortium of *Chlorella* sp., *Nannochloropsis* sp., *Scenedesmus bijugatus*, *Chlamydomonas reinhardtii*, and *Oscillatoria*,^115^ respectively, when using 75% diluted municipal wastewater.

In addition to these methods, Madeira et al.^116^ highlighted that combining phytohormones with abiotic stress factors through two-stage cultivation is a promising strategy to enhance lipid accumulation in microalgae further. However, when developing such strategies, it is crucial to ensure high biomass productivity simultaneously. Molecular tools hold significant potential for improving microalgae’s tolerance to abiotic stress, thereby increasing biomass productivity under extreme conditions and enhancing lipid yields.^116^ Nevertheless, challenges remain, particularly regarding the economic feasibility of large-scale processes, including the high energy demands of the biomass drying step. Emerging methods for wet lipid extraction, such as supercritical CO_2_ extraction,^117^ microwave-assisted lipid extraction,^118^ and other innovative techniques,^119^ offer potential solutions by eliminating the need for drying.

Briquette production is gaining attention as a straightforward and versatile approach to energy recovery. This method combines biomass with various wastes, such as manure, microalgae, plastics, sludge, and food waste, to produce briquettes with a HHV ranging from 13.2 to 24.9 MJ kg^–1^. The simplicity and availability of raw materials make this technique particularly attractive.^120^

Anaerobic digestion performance varies widely depending on algal composition and operational strategies, such as mono- or codigestion. Despite these variations, energy yields can reach up to 5.67 kWh·kg^–1^ VTS, making AD a competitive route.

HTL remains a promising technique for producing liquid biofuels from various microalgae species. While operational parameters strongly influence yield and composition, further research aims to optimize process conditions and improve fuel upgrading into renewable diesel and aviation fuels.

Thus, upgrading processes for crude bio-oil can be implemented to meet current fuel standards. Using catalysts effectively reduces undesirable heteroatoms in bio-oil, such as oxygen (O), N, and sulfur (S). These processes involve techniques such as hydrodeoxygenation (HDO), hydrodenitrogenation (HDN), and hydrodesulfurization (HDS) under high hydrogen pressures (10–20 MPa) and moderate temperatures (250–450 °C).^125^ Catalysts like zeolites, such as Zeolite Socony Mobil (ZSM-5), where “5” represents the pore size in angstroms,^126^ and metals like nickel (Ni), cobalt (Co), palladium (Pd), and platinum (Pt), supported on materials like alumina, are commonly used.^127^ Bimetallic catalysts, such as Ni-Mo or Co-Mo combinations, have enhanced the efficiency of HDO, HDN, and HDS reactions.^128^

Additionally, research has highlighted the valorization of HTL coproducts to improve the process’s viability and sustainability.^129^ Strategies such as using AP as a medium for algal cultivation, reducing biomass moisture, and increasing heat recovery within the reactor have proven effective in mitigating the environmental impact of the process.^109^ For instance, the recirculation of AP in microalgae cultivation^130–132^ and its utilization in alternative valorization pathways, such as supercritical fluid gasification for biohydrogen (bio-H_2_) production,^133,134^ are being actively explored.

Due to the high ash content, the HTL solid phase may have limited potential for applications such as hydrochar, catalyst support,^135^ or as a biofertilizer for soil amendment.^136^ However, alternative uses are being investigated, such as its incorporation into asphalt production, as suggested by Lorentz et al.^137^ The gaseous phase, primarily composed of CO_2_, can also be used in microalgae cultivation,^7^ aligning with the principles of a circular bioeconomy.

HTC produces solid biofuels with enhanced energy density and handling properties. Hydrochar HHVs vary with biomass and conditions, and ongoing research seeks to tailor its applications.^138^

To facilitate energy-based comparison among these routes, estimated outputs in kWh·kg^–1^ of dry biomass (or per kg of volatile solids, when appropriate) are provided and discussed in Table 3.

**Table 3:** Estimated Energy Recovery from Wastewater-Grown Microalgae via Different Routes^a^

Conversion route	Reported yield	Energy unit in literature	Estimated energy output (kWh·kg^–1^)	Basis
Lipid extraction (biodiesel)	8.9–10% lipid/HHV ≈ 37 MJ kg^–1^ ^108^	MJ kg^–1^ (lipid)	0.33–0.37	Dry biomass
Briquetting	13.2–24.9 MJ kg−1 ^122^	MJ kg^–1^	3.67–6.92	Dry biomass
Anaerobic digestion	0.20–0.57 m^3^ CH_4_ kg VTS−1 ^107^	m^3^ CH_4_ kg^–1^	1.99–5.67	VTS
Hydrothermal liquefaction	37.4–44.4% bio-oil, HHV ≈ 38.1 MJ kg−1 ^128^	Yield (%) and HHV (MJ kg^–1^)	3.93–4.66	Dry biomass
Hydrothermal carbonization	60–77% hydrochar, HHV = 16.9–28.32 MJ kg−1 ^113^	Yield (%) and HHV (MJ kg^–1^)	2.81–6.05	Dry biomass

a 1 MJ = 0.2778 kWh.

HTL presents a bio-oil yield ranging from 37.4% to 44.4%, with a HHV of approximately 38.1 MJ kg^–1^, resulting in an estimated energy output of 3.93 to 4.66 kWh per kg of dry biomass. HTC yields hydrochar with HHVs from 16.9 to 28.32 MJ kg^–1^ and mass yields between 60% and 77%, corresponding to approximately 2.81 to 6.05 kWh per kg of dry biomass. AD generates 0.20 to 0.57 m^3^ CH_4_ per kg of VTS, considering an HHV of 35.8 MJ m^–3^ for methane. This translates to an energy output of approximately 1.99–5.67 kWh per kg of VTS.

### 4.2 Agricultural Use

As in many developing countries, Brazil’s agriculture is critical due to its significant social, environmental, and economic importance. Globally, Brazil ranks as the third-largest agricultural producer and the second-largest food exporter. The country also possesses the world’s largest tropical forest, where deforestation driven by agricultural expansion contributes to more than half of its annual GHG emissions.^136^ Consequently, there is an urgent global need to promote low-GHG agricultural development that ensures food security while aligning with sustainable practices, particularly in agriculture-dependent nations like Brazil.

Biofertilizers offer a promising solution by reducing reliance on nonrenewable natural resources, such as phosphorus, and mitigating GHG emissions by avoiding energy-intensive processes like the Haber–Bosch method for nitrogen fertilizer production. Research on developing biofertilizers from biomass derived from wastewater treatment has intensified, given the potential of microalgae biotechnology to enable multiple production pathways. Studies on biofertilizers have yielded promising results. In this context, the research group has focused on integrated solutions within the water-food-energy nexus, advancing the development of bioproducts for agricultural applications.

#### 4.2.1 Establishment of Microalgae Biofilm in Soil

Castro et al.^110^ evaluated GHG emissions, ammonia (N-NH_3_) volatilization, and the growth of *Pennisetum glaucum* under the influence of a microalgae biofilm. Their findings demonstrated that establishing a microalgae biofilm in the soil reduced nitrogen losses through N-NH_3_ volatilization (4.63% loss with the biofilm compared to 18.98% with urea). They increased both soil organic matter content and cation exchange capacity. However, the biofilm treatment resulted in higher CO_2_ and nitrous oxide (N_2_O) emissions, which were 1.8 and 5.6 times greater, respectively, than those observed with chemical fertilizer. Additionally, chemical fertilizer outperformed the biofilm treatment in plant dry matter production, yielding 1.2 times more biomass.

When modeling the environmental impacts associated with producing 1 kg of N from microalgae biomass and comparing it to the production of the same amount of N from commercial urea fertilizer, Souza et al.^21^ reported that biofertilizer production caused a significantly higher environmental impact, 90% greater, particularly in the climate change category. They highlighted the importance of including the cultivation stage in LCA to understand the benefits of microalgae-based products comprehensively. Alternatively, they suggested that emissions from the cultivation phase should not be fully attributed to the biofertilizer but instead account for avoided products, such as potable water and nutrients, particularly when wastewater is used in cultivation rather than synthetic media.

Lorentz et al.^31^ investigated biomass cultivated in HRAP with wastewater from cattle farming as a substitute for conventional chemical fertilization in acidic soil for cultivating *Urochloa brizantha* cv. Marandu grass. Their results showed that the biological fertilization treatment achieved the highest dry matter content (23%) compared to the control (no fertilization) at 21%. For crude protein and ash content, chemical fertilization exhibited the highest values (12.81% and 8.71%, respectively), followed by biological fertilization (10.27% and 7.17%, respectively). Regarding neutral detergent fiber content, the chemical treatment achieved the lowest value (62.55%), followed closely by the biological treatment (62.85%).^31^

While no significant differences were observed in soil C and N contents between chemical and biological fertilization treatments, higher S and iron (Fe) contents were recorded in the biological treatment. Enzyme activities also differed: α-glucosidase and leucine-aminopeptidase activities were higher in the biological treatment, whereas acid phosphatase, *N*-acetylglucosaminidase, and sulfatase activities were greater in the chemical treatment. Interestingly, β-glucosidase activity was highest in the control treatment.^137^ Similarly, Feng et al.,^138^ comparing organic and conventional chemical fertilization, observed increased α-glucosidase activity with organic fertilization. These enzymes are crucial in enhancing soil mineralization and decomposition of organic matter.

Scaling up studies provided valuable insights into the previously limited understanding of the effects of algal biomass application on soil enzyme activity, advancing knowledge of its potential benefits and trade-offs in agricultural practices.

#### 4.2.2 Phosphate and Nitrogen Biofertilizers

The promising results of using microalgae biomass in soil have highlighted the need for research into granulating and pelletizing this biomass as a biofertilizer. This approach could improve scalability and align biotechnology with commercial standards for storage and logistics. Castro et al.^139^ studied the absorption of P by plants fertilized with triple superphosphate (TSP) enriched with varying proportions (5%, 10%, 15%, 20%, and 30%) of microalgae biomass cultivated in wastewater from the meat processing industry. The results showed that P diffusion in the soil decreased at higher biomass concentrations in the biofertilizer. In comparison, at lower concentrations, P diffusion occurred at a rate the plant could not assimilate.

In a related study, Castro et al.^140^ evaluated the environmental impacts of producing 1 kg of this biofertilizer at the optimal proportion for P assimilation by plants, as determined in the previous study. They compared these impacts with those of producing 1 kg of TSP fertilizer. The microalgae biofertilizer exhibited greater environmental impacts than TSP across all analyzed categories, particularly for climate change (75%) and terrestrial ecotoxicity (87%). The primary contributor to these impacts was the drying stage, where the microalgae biomass, harvested with a moisture content of 99% via gravitational settling, required significant energy. Additionally, granulating the biomass presented challenges; proportions above 30% biomass resulted in granules lacking sufficient shape and rigidity, leading to disintegration (unpublished data).

While the TSP-based biofertilizer with 12% microalgae biomass demonstrated technical feasibility, the environmental analysis indicated its limitations compared to commercial TSP under the defined analysis criteria. Future studies should aim to (i) include differences in plant productivity resulting from the application of both fertilizers, (ii) exclude the environmental impacts from wastewater treatment, as this process is mandatory and not optional, and (iii) propose energy-efficient methods for drying microalgae biomass.

Pereira et al.^112^ explored the technical performance of a pelletized organomineral biofertilizer composed of synthetic fertilizer (urea) mixed with varying proportions of microalgae biomass (5%, 15%, 30%, 40%, and 50%) cultivated in wastewater. They assessed the biofertilizer’s effect on corn (*Zea mays* L.) productivity, N assimilation, and N-NH_3_ volatilization losses over time. The highest N recovery rates were achieved in treatments with 15% and 30% microalgae biomass (54.23% and 53.18%, respectively). The highest plant productivity was observed with 50% microalgae biomass, potentially due to micronutrients present in the biomass. For example, boron concentrations increased linearly with rising biomass proportions (*p* < 0.05).

Regarding volatilization losses, N-NH_3_ emissions were detected 3 days after biofertilizer application across all treatments, with the highest cumulative losses observed at 40% microalgae biomass. The microalgae biomass appeared to promote volatilization, possibly due to the NaOH used during harvesting, which increased the biofertilizer’s pH. This finding underscores the need for more efficient harvesting methods that avoid chemical additives, which could compromise the biofertilizer’s technical performance. The physicochemical properties of the fertilizer pellets also influenced results, particularly in terms of N availability, as microalgae biomass coated the urea crystals.

In an LCA, Pereira et al.^141^ found that environmental impacts increased with the concentration of microalgae biomass in the biofertilizer. The primary contributors were NaOH use during the harvesting stage (as corroborated by Souza et al.^21^ in Section 2) and energy consumption during cultivation and drying. However, modeling an optimized scenario with the best-performing biofertilizer (15% microalgae biomass) and substituting NaOH chemical coagulation with GS reduced impacts by 24% and 52% compared to the nonoptimized scenario and synthetic fertilizer.

The findings emphasize the need for future research to focus on (i) Integrating biofertilizer production with efficient harvesting technologies that eliminate chemical inputs, (ii) Developing cultivation strategies to enhance microalgae productivity, and (iii) Quantifying the benefits to soil quality derived from microalgae-based biofertilizer application.

#### 4.2.3 Microalgae Biomass as a Biostimulant

Silva et al.^111^ evaluated the potential of microalgae biomass derived from meat processing wastewater as a biostimulant for corn cultivation (*Zea mays* L.). The study tested various biomass concentrations (0.25%, 0.5%, 1%, and 2% w/w) in soils from the A horizon (0–20 cm) and B horizon (20–40 cm). Results showed that the 2% biomass application enhanced soil biostimulation indicators such as basal respiration, microbial biomass C, and soil enzymatic activity. However, it was not the most effective in promoting plant growth. In contrast, applying 0.55% biomass led to the highest plant productivity. This discrepancy may be attributed to sodium (Na) in the biomass, which likely caused toxicity at higher application rates.

Castro et al.^110^ and Pereira et al.^112^ have similarly noted that sodium is introduced into the soil when microalgae biomass is harvested using NaOH in the chemical flocculation. The sodium concentration in the soil increased proportionally with the biomass application rate, leading to elevated Na levels in the plants. In this study, higher biomass doses, such as the 2% application, likely caused sodium toxicity, which inhibited plant growth. This observation highlights the need to optimize the biomass concentration to maximize biostimulant potential while minimizing the risks associated with Na accumulation in soil and plants.

Moreover, as emphasized in Silva et al.,^142^ the biomass harvesting phase, particularly the use of NaOH as a coagulant, was identified as a critical factor contributing to the overall process’s C footprint. These findings underscore the importance of developing sustainable harvesting methods that reduce sodium content in microalgae biomass, thereby enhancing its efficacy as a biostimulant while mitigating environmental impacts.

In summary, while microalgae biomass shows promise as a biostimulant, optimizing the concentration and addressing the challenges of Na-induced toxicity is crucial for achieving sustainable and effective agricultural applications. Additionally, reducing the environmental burden of the biomass harvesting process is key to improving the overall sustainability of microalgae-based biostimulants.

#### 4.2.4 Future Perspectives on the Agricultural Use of Wastewater-Grown Algal Biomass

The results summarized in Table 4 highlight key findings, and the prospects for each agricultural application are thoroughly discussed.

**Table 4:** Methods of Application, Predominant Species, Dosages, and Key Outcomes of Wastewater-Grown Microalgae Biomass Utilization in Agriculture over 15 Years of Research^a^

Application method	Predominant species	Dose	Main results	Reference
Microalgae biomass cultivated in agro-industrial wastewater for the growth of millet (*Pennisetum glaucum* L.).	*Chlorella vulgaris*	(i) 120 kg ha^–1^ of N provided by the algal biomass; (ii) 120 kg ha^–1^ of N provided by conventional urea; and (iii) a control lacking supplement with any fertilizer type.	The application of microalgae biomass increased the soil’s nitrogen content, cation exchange capacity, and organic matter.	^110^
			Significant differences were observed for shoot dry matter mass and nitrogen content in the plants from both treatments where nitrogen sources were applied. All treatments differed in leaf dry matter mass, with the urea treatment increasing the most.	
Microalgae biomass cultivated in wastewater from cattle farming for the cultivation of *Urochloa brizantha* cv. Marandu	*Scenedesmus* sp.	(i) 40 kg ha^–1^ per cut of the mixture N:P:K 20:0:20; (ii) applying microalgae biomass corresponding to 40 kg N ha^–1^ at each cut, calculated based on its N content (TKN content), totalizing 24 m^3^ ha^–1^ of wet biomass; and (iii) did not receive any fertilizer.	The biological treatment had similar plant productivity to the treatment with conventional chemical fertilizer. Regarding nitrogen utilization efficiency and nitrogen recovery, the chemical treatment showed a statistical difference compared to microalgae biomass.	^31^
Granular biofertilizer produced from microalgae cultivated in agro-industrial wastewater to grow millet (*Pennisetum glaucum* L.).	*Chlorella vulgaris*	Biofertilizer with MB proportions of 5, 10, 15, 20 and 30% (w.w^–1^). TSP without the addition of biomass was used as a control.	In a greenhouse, the P content presented a significant difference for the tests carried out with TSP and TSP + 12% MB fertilizer, the latter of which provided a higher plant recovery rate.	^139^
Tablet biofertilizers produced from microalgae cultivated in agro-industrial wastewater for the growth of corn (*Zea mays* L.).	*Chlorella vulgaris*	Biofertilizer with MB proportions of 5, 15, 30, 40, and 50% (w.w^–1^). Urea without the addition of biomass was used as a control.	From 25% of MB, there is no increase in N absorbed by plants, while the volatilization of N-NH_3_ grows with the increase in MB.	^112^
Microalgae biomass cultivated in agro-industrial wastewater applied to corn growth (*Zea mays* L.).	*Chlorella vulgaris*	Doses of 0.25%, 0.5%, 1%, and 2% biomass were applied in soils from (i) Horizon A, taken at a depth between 0 and 10 cm, and (ii) Horizon B, taken at a depth between 20 and 40 cm.	The result that provided the higher plant shoot dry matter mass was by applying 0.55% biomass in both soils.	^111^

a N = nitrogen; P = phosphorus; K = potassium; TKN = Total Kjeldahl nitrogen; MB = Microalgae biomass; TSP = Triple Superphosphate; N-NH_3_ = Ammonia.

Microalgae biomass continues to be a promising alternative for sustainable and organic agriculture. Samoraj et al.^143^ identified this biomass as a strategic raw material for producing biobased fertilizers, especially in light of the ongoing natural resource crisis. Its rich composition of macro- and micronutrients and bioactive compounds offers significant potential to enhance agricultural productivity. Moreover, its application can improve soil fertility and reduce reliance on synthetic fertilizers, thereby contributing to the sustainability of agricultural systems.^143^

Microalgae-based biofertilizers have been shown to influence soil microbiota, enhancing both productivity and quality of *P. tenuifolia*.^144^ This suggests that integrating microalgae with microbial inoculants could effectively minimize chemical fertilizer use while promoting sustainable agriculture.^144^ Su et al.^144^ demonstrated that substituting 20% of chemical fertilizers with microalgae not only improved soil quality but also significantly increased organic matter content by 15.68% (*p* < 0.05). Additionally, the addition of microalgae altered the bacterial composition in the plant rhizosphere, reducing the relative abundance of *Cladosporium* by 33.33% and 57.93%, while increasing *Chloroflexi* abundance by 31.06% and 38.27% under 20% and 40% reductions in chemical fertilizers, respectively.^144^

Other studies have further explored the potential of microalgae in agriculture. For instance, Viegas et al.^145^ investigated the biostimulatory effects of microalgae cultivated in wastewater, focusing on seed germination rates of watercress (*Nasturtium officinale*) and wheat (*Triticum aestivum*) at concentrations of 0.2 and 0.5 g L^–1^. Their findings positively impacted seed germination.^145^

Similarly, Navarro-López et al.^146^ evaluated the biostimulatory potential of *Scenedesmus obliquus* using different biomass processing techniques. Their study assessed bioactivity through seed germination tests in watercress, induction of adventitious roots in mung bean (*Vigna radiata*), and cotyledonary root growth and tissue expansion in cucumber (*Cucumis sativus*). The unprocessed biomass exhibited a germination rate 40% higher than the control, while a treatment combining ultrasound, enzymatic hydrolysis, and centrifugation at 0.5 g L^–1^ increased adventitious root induction by 67.9%.^146^

Applying microalgae-based biofertilizers offers numerous advantages, including replacing chemical fertilizers, preserving soil health, and preventing water contamination while contributing to C sequestration. These biofertilizers can be tailored to release specific nutrients and biopesticides, aiding pest control and boosting agricultural productivity. To facilitate the adoption of these technologies, establishing integrated biorefineries, developing supportive public policies, and increasing awareness of their environmental benefits are essential. These efforts will help drive the transition toward a sustainable circular bioeconomy.^147,148^

### 4.3 Carotenoids and Fatty Acids

In addition to bioenergy and biofertilizers, numerous high-value bioproducts can be derived from microalgae’s primary and secondary metabolites.^4^ For instance, lipids can be converted into polyunsaturated fatty acids (PUFAs), carbohydrates serve as potential sources of bio-H_2_, proteins can be transformed into biopolymers, and pigments can yield high concentrations of carotenoids. In this context, the research group has expanded its scope to explore microalgae-based biorefineries to enhance production cost efficiency.

Carotenoids, bioactive pigments produced by algae, play a key role in energy capture, supporting photosynthesis and cellular survival under stress conditions.^149^ These pigments also hold significant commercial value.^150^ Braga et al.^109^ evaluated carotenoid production using blends of domestic wastewater and paint booth wastewater, hypothesizing that nitrogen limitation stress caused by adding paint booth wastewater would enhance carotenoid accumulation in microalgal biomass. In a bench-scale experiment, eight treatments were tested, ranging from 0% to 100% paint booth wastewater (v/v) (0%, 1%, 5%, 10%, 25%, 50%, 75%, and 100%). Treatments with 25% and 50% paint booth wastewater achieved the highest carotenoid concentrations, close to 3 mg L^–1^, likely due to an improved nutrient ratio in the cultivation medium (C/N ratio).^109^

Although cultivation conditions influence carotenoid production and accumulation,^151^ higher concentrations of paint booth wastewater, and consequently reduced nutrient availability, resulted in lower algal biomass growth and total carotenoid productivity,^109^ contrary to the initial hypothesis. These findings suggest that optimizing nutrient availability is more effective for improving carotenoid accumulation than solely inducing stress conditions. Like lipid accumulation (Section 4.1.1), enhancing biomass productivity under favorable cultivation conditions is a more practical and replicable strategy.

In another study, Ferreira et al.^74^ analyzed carotenoid productivities in different cultivation reactors, HRAP and HS, using domestic wastewater and agro-industrial wastewater from the meat processing industry. The study primarily identified β-carotene and lutein as the main carotenoids. Cultivation with domestic wastewater resulted in higher lutein concentrations, with 55.6 μg g^–1^ in the HS reactor and 52.6 μg g^–1^ in the HRAP reactor. Conversely, agro-industrial wastewater produced more β-carotene, with a maximum concentration of 32.8 μg g^–1^. These findings suggest that while the reactor type had minimal impact on carotenoid concentrations, the type of wastewater significantly influenced the specific carotenoid produced by the microalgae.

From an LCA, the authors concluded that producing 1 kg of carotenoids had significant environmental impacts, particularly in terms of human carcinogenicity and health risks, primarily due to the use of ethyl acetate in the extraction process and electricity consumption.

Silva et al.^152^ evaluated the cultivation of microalgae in a medium composed of industrial and domestic wastewater, identifying a lipid profile dominated by saturated fatty acids. Among these, palmitic acid (C16:0) stood out, accounting for 36.9% of the total lipid fraction. Unsaturated fatty acids were also quantified, including linoleic acid (C18:2) at 17.8%, oleic acid (C18:1) at 15.2%, and α-linolenic acid (C18:3) at 10%. Other relevant saturated fatty acids included pentadecanoic (8.5%), stearic (5.2%), and arachidic (2.8%) acids. The authors highlighted the energy potential of these compounds, particularly C16:0, which can be converted into aliphatic hydrocarbons such as heptadecane (C17) through HTL, a typical component of renewable diesel.

Similar results were observed by Pereira et al.,^39^ who also used a blend of domestic sewage and effluent from the juice industry, focusing on the effect of the carbon-to-nitrogen (C/N) ratio on microalgal cultivation performance and biomass biochemical composition. The study found that a C/N ratio of 30.67 promoted the accumulation of carbohydrates (30.07%) and lipids (26.39%), while a lower C/N ratio of 7.52 favored protein synthesis (33.00%). The lipid profile included saturated fatty acids (SFA), monounsaturated fatty acids (MUFA), and polyunsaturated fatty acids (PUFA), with palmitic acid (C16:0) once again being the most abundant (31.11% to 39.77%), followed by oleic acid (C18:1, 24.4–32.16%), linoleic acid (C18:2, 9.49–17.83%), and linolenic acid (C18:3, 5.94–16.71%). The study demonstrated that high C/N ratios tend to favor the synthesis of short-chain fatty acids such as C16:0.

Carotenoids, particularly β-carotene and lutein, reached their highest concentrations at a C/N ratio 30.67, with 12.81 μg g^–1^ and 507.97 μg g^–1^ of dry biomass, respectively.^39^ These pigments are critical for photosynthesis and possess significant commercial value in the food and pharmaceutical industries. Nitrogen-limited conditions associated with high C/N ratios appeared to favor carotenoid accumulation. Overall, these findings highlight the importance of controlling the C/N ratio in microalgae cultivation to optimize wastewater treatment while maximizing the production of high-value biocompounds.

## 5 Challenges and Opportunities

The application of microalgae biotechnology, particularly cultivation through wastewater treatment, offers a promising solution to several global environmental challenges. These include expanding wastewater treatment in small towns and isolated communities, reducing CO_2_ emissions across various sectors, diversifying local energy portfolios, and decreasing reliance on chemical fertilizers and fossil fuels. Figure 3 highlights the numerous integration opportunities within a microalgae biorefinery focused on resource recovery from wastewater.

**Figure 3:**
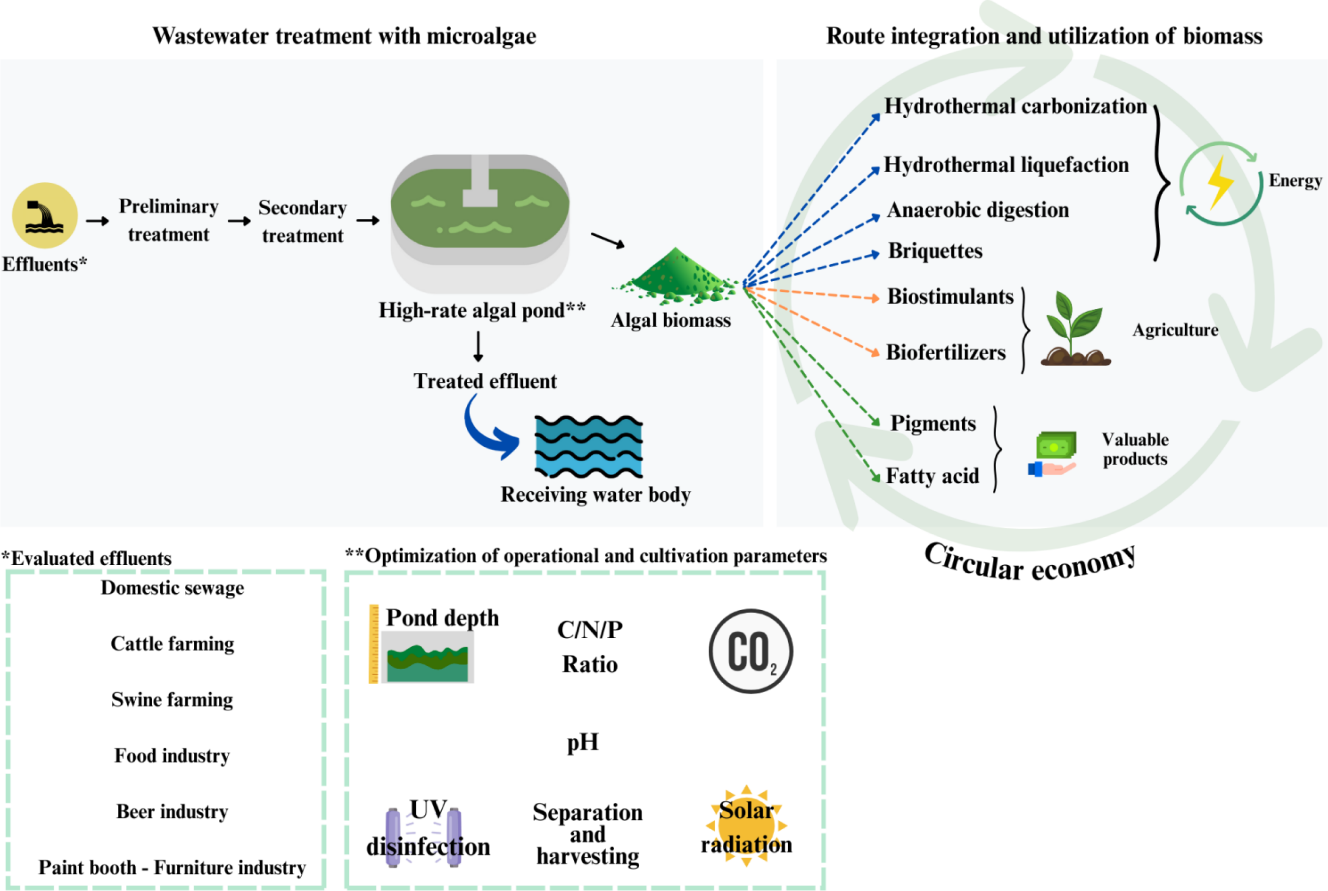
Integration opportunities within a microalgae biorefinery focused on resource recovery from wastewater.

Fifteen years of research at the Federal University of Viçosa have significantly advanced the integration of algal biomass production with wastewater treatment, consolidating knowledge in several key areas:

•Despite variability in wastewater characteristics, the composition of the resulting biomass is directly influenced by the cultivation medium’s properties. For example, protein predominates in biomass grown in wastewater with low C/N ratios.

•Design and operational parameters (pond depth, CO_2_ addition, and UV radiation) are critical for optimizing microalgae production and minimizing land requirements for HRAPs.

•Biomass harvesting mechanisms can significantly impact biomass composition, often limiting its suitability for specific end-use applications.

•Energy-efficient alternatives for processing wet biomass, such as AD and hydrothermal technologies, are economically and environmentally attractive due to reduced drying requirements.

•Microalgae biomass is a valuable feedstock for nitrogen and phosphate biofertilizers, offering a vital pathway for nutrient recycling from wastewater.

In addition to these achievements, new opportunities have emerged from the research findings:

•**Blended Wastewater**: Blending wastewater streams to increase the C/N ratio, typically low in domestic wastewater, can enhance biomass productivity and stimulate lipid and carbohydrate accumulation. This approach may be more cost-effective and operationally feasible than CO_2_-rich gases. These hypotheses warrant further technical and environmental validation through LCA.

•**Emerging Contaminants**: Microalgae can effectively remove emerging contaminants such as pharmaceuticals, pesticides, and microplastics, a topic under investigation by several research groups globally. Establishing the relationship between removal mechanisms and design criteria is critical for scaling microalgae technologies. Furthermore, whether removal occurs via adsorption or assimilation, the impact of these contaminants on the biomass’s downstream applications must be evaluated.

•**Biomass Harvesting**: Among the emerging approaches, electrocoagulation stands out for combining high biomass recovery efficiency with the potential for additional functionalities, such as hydrogen coproduction and mineral enrichment of the biomass for agricultural applications. The use of electrodes composed of metals essential for plant nutrition, such as iron, zinc, and magnesium, adds value to the harvested biomass by enhancing its suitability as a natural biofertilizer. These features make electrocoagulation a particularly attractive alternative from the standpoint of sustainability and process multifunctionality.

•**Solar Energy for Biomass Harvesting**: It is essential to explore solar energy as a cost-effective drying method. Defining design parameters for solar drying beds downstream of HRAPs could enhance their adoption in wastewater treatment plants.

•**Co-digestion for CH**_**4**_ **Optimization**: Combining complementary feedstocks can optimize methane production by correcting the C/N ratio in AD. Additionally, the two-stage digestion of microalgae biomass could be explored as a novel approach for bio-H_2_ production.

•**Byproducts from HTL**: Utilizing HTL byproducts, such as water-soluble fractions and solid residues, is critical for the process’s economic and environmental feasibility. Advancing bio-oil upgrading technologies remains a priority.

•**Biostimulant Compounds**: Promoting the accumulation of biostimulants, including phytohormones, EPS, and amino acids, along with their extraction, can open new opportunities for microalgae applications.

•**High-Value Products**: Advancing the production of other high-value products, such as pigments and polymers, offers exciting prospects. However, further research on public acceptance is crucial, particularly for products intended for human consumption.

## 6 Concluding Remarks

Over 15 years of research, the integration of microalgae-based systems with wastewater treatment has proven to be a promising strategy for environmental remediation. The studies conducted at the Federal University of Viçosa for wastewater treatment have demonstrated that HRAPs, HS and PBRs can effectively remove nutrients and organic matter from a variety of effluents (domestic and agro-industrial), while simultaneously generating biomass rich in proteins, carbohydrates and lipids.

Among the tested configurations, hybrid systems, comprising HRAPs coupled with biofilm reactors, stood out not only for their higher biomass productivity and reduced dependence on CO_2_ supplementation, but also for their operational advantages. These include improved biomass recovery efficiency and greater harvesting feasibility, as the adhered biomass can be manually or mechanically scraped, reducing reliance on energy-intensive separation techniques. When compared to conventional HRAPs, HSs achieved higher biomass concentrations, simplified post-treatment processes, and offered greater adaptability to climatic variations through design adjustments such as seasonal inclination angles of biofilm panels. Moreover, low-cost coagulants and autoflocculation strategies further contributed to enhancing biomass separation efficiency in a technically and economically viable manner.

From an environmental perspective, LCAs have revealed that higher biomass recovery efficiencies, particularly in hybrid systems with attached biomass, are crucial for reducing impacts. Strategies that favored attached growth, such as hybrid systems, resulted in reduced energy demand during collection and subsequent processing and led to reductions of up to 40% in categories such as global warming potential, fossil fuel depletion, and freshwater eutrophication. These insights highlight that environmental sustainability in algae-based systems is not only linked to reactor performance, but also to collection logistics, among other factors.

In parallel, the studies explored various operational strategies to optimize nutrient removal and biomass production, including UV predisinfection, shade management, HRAP depth adjustments, and carbon supplementation. Carbon limitation, a common constraint in domestic wastewater, was successfully mitigated both by direct CO_2_ injection (including flue gas recycling) and by blending effluents with high organic loads, such as those from paint booths and breweries. These approaches improved the C/N/P balance of the medium, increased algal productivity, and increased overall treatment efficiency.

Furthermore, modulating HRAP depth proved to be an effective strategy for balancing productivity and reducing the required area without compromising system performance. Shallower reactors supported nutrient uptake by biomass, whereas increased depths, made possible through methods like predisinfection, sustained system efficiency despite reduced light exposure. Further investigating the treatment of pig farm wastewater, the studies demonstrated the systems’ ability to remove heavy metals, such as copper and zinc, while also highlighting their adverse effects on the removal of nutrients, especially nitrogen.

Finally, the study demonstrated the feasibility of multiple valorization routes for the biomass obtained, including its use as biofertilizer, briquettes for direct combustion, AD for biogas, and HTL for bio-oil production. Although some routes, such as lipid extraction for biodiesel, were limited by low lipid content and high drying energy demands, alternatives like wet biomass valorization proved more promising from both environmental and energy perspectives. The versatility of the biomass, particularly its high protein content, also points to potential applications in animal nutrition and as a base for agronomic bioproducts. Thus, the research reinforces the potential of integrating wastewater treatment with resource recovery through algal biotechnology, advancing decentralized, circular, and sustainable solutions for sanitation and bioeconomy challenges.

## Supplementary Material



## Notes

Average biochemical composition of algal biomass produced in major types of wastewater (Figure S1); Hydrothermal liquefaction byproduct yields as a function of algal biomass ash content (Figure S2) (PDF)

## Vocabulary:

AD: anaerobic digestion
AP: aqueous phase
BOD: biochemical oxygen demand
BR: biofilm reactor
C: carbon
COD: chemical oxygen demand
CH_4_: methane
Co: cobalt
CO_2_: carbon dioxide
Cu: copper
DO: dissolved oxygen
EPS: extracellular polysaccharides
Fe: iron
GHG: greenhouse gas
GS: gravitational settling
H: hydrogen
HDN: hydrodenitrogenation
HDO: hydrodeoxygenation
HDS: hydrodesulfurization
HRAP: high-rate algal ponds
HRT: hydraulic retention time
HS: hybrid system
HTC: hydrothermal carbonization
HTL: hydrothermal liquefaction
K: potassium
LCA: life cycle assessment
N: nitrogen
NAOH: sodium hydroxide
Ni: nickel
N-NH_4_^+^: ammonia nitrogen
N-NO_3_^–^: nitrate
N_2_O: nitrous oxide
O_2_: oxygen gas
PAR: photosynthetically active radiation
PBR: photobioreactor
Pd: palladium
P: phosphorus
Pt: platinum
SDGs: Seventeen Sustainable Development Goals
ST: settling tank
TC: tannin coagulation
TOC: total organic carbon
TSP: triple superphosphate
UASB: upflow anaerobic sludge blanket
UN: United Nations
UV: ultraviolet
VSS: volatile suspended solids
Zn: zinc
ZSM: Zeolite Socony Mobil
