# Supplementary material for: Microalgae-Based Wastewater Treatment and Biomass Valorization: Insights, Challenges, and Opportunities from 15 Years of Research
Source: ACS Omega. 2025 Oct 17;10(42):49273–99. doi: 10.1021/acsomega.5c04364 (PMC12572981; doi:10.1021/acsomega.5c04364)
Supplement: Supplementary file 1 [file ao5c04364_si_001.pdf]

Supporting Information

Microalgae-Based Wastewater Treatment and Biomass Valorization: Insights, Challenges, and Opportunities from 15 Years of Research

Maria Lúcia Calijuri[a*], Eduardo de Aguiar do Couto[b], Paula Peixoto Assemany[b], Vinicius José Ribeiro[a], Juliana Ferreira Lorentz[a], Jackeline de Siqueira Castro[a], Letícia Rodrigues de Assis[a], Adriana Paulo de Sousa Oliveira[a], Alexia Saleme Aona de Paula Pereira[a], Bianca Barros Marangon[a], Iara Barbosa Magalhães[a], Thiago Abrantes Silva[a], Jéssica Ferreira[a], Matheus Quintão Braga[a], Rafael Carvalho Nogueira da Gama[a], Bruno Silva Henriques[a]

[a]Post-Graduate Programm in Civil Engineering, Center for Advanced Research in Microalgae, Federal University of Viçosa (*Universidade Federal de Viçosa*), University Campus, zip code 36.570-900, Viçosa, Minas Gerais, Brazil.

[b]Post-Graduate Programm in Environmental Engineering, Environmental Engineering Department, Federal University of Lavras (*Universidade Federal de Lavras*), University Campus, zip code 37203-202, Lavras, Minas Gerais, Brazil.

***Corresponding author.** Address: PH Rolfs Avenue, no number - Civil Engineering Building, Room 320 - University Campus - ZIP Code 36.570-900 - Viçosa, Minas Gerais, Brazil; e-mail: calijuri@ufv.br; phone: +553136126136

In Figure S1, the biochemical composition of the biomass cultivated under different wastewater type is presented. The results are a summary of different studies conducted by the research group.

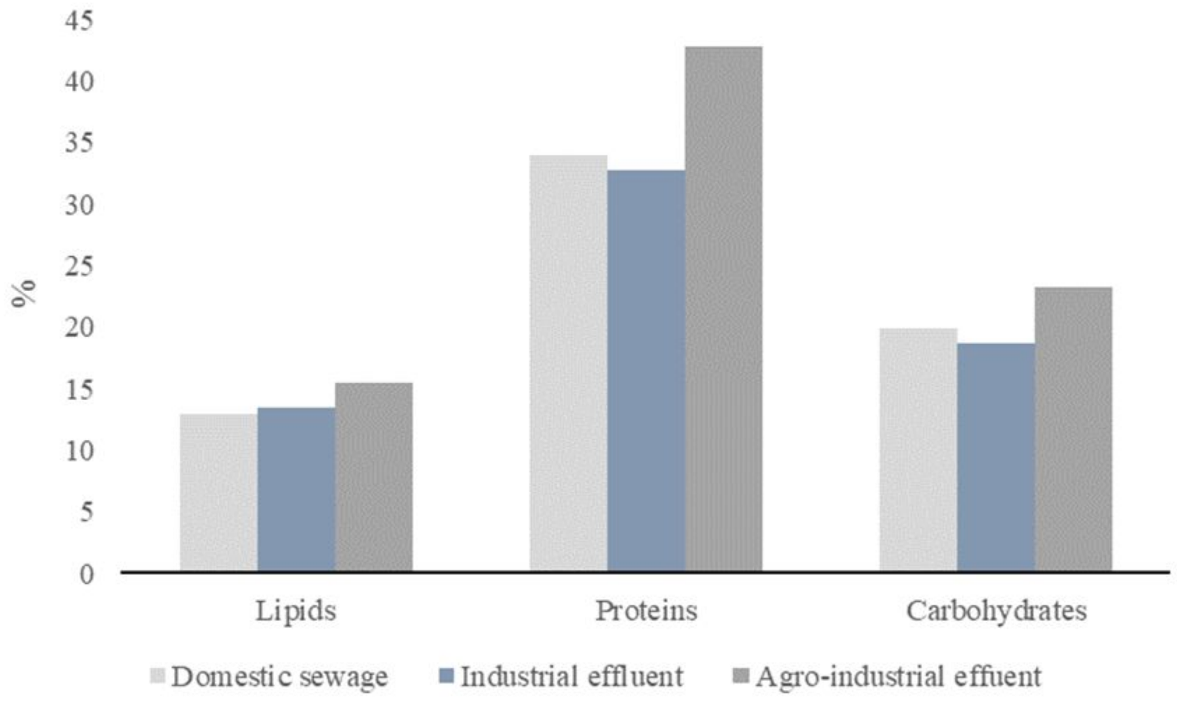

Figure S1. Average biochemical composition of algal biomass produced in major types of wastewater (agro-industrial wastewater includes the following types: swine farming, meat processing, cattle farming; and industrial wastewater: food industry, beer industry and painting booth for the furniture industry).

For the biomass produced in wastewater treatment in HRAPs, the Figure S2 presents different ash contents on bio-oil yield, according to Couto (2016)[1].

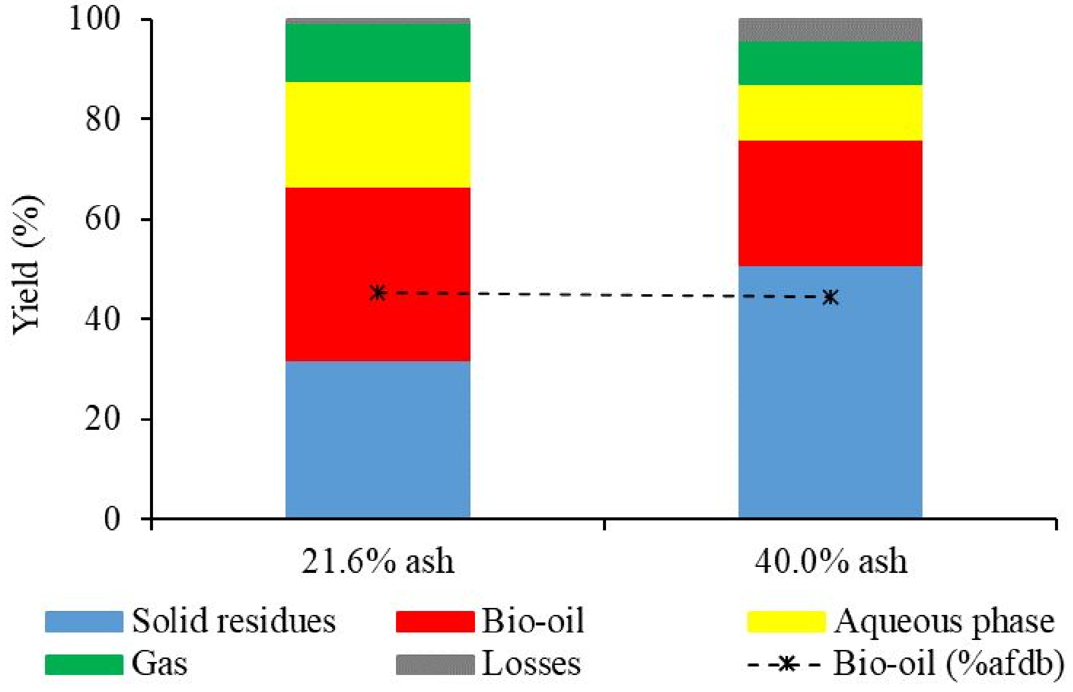

Figure S2. Yield of hydrothermal liquefaction (HTL) by-products as a function of different ash contents in algal biomass (afdb = ash free and dry basis).

Reference:

(1) Couto, E. de A. *Biomass Production in High-Rate Algal Ponds with Different Depths and Its Utilization for Energy Generation via Hydrothermal Liquefaction*; Ph.D. Thesis, Federal University of Viçosa, 2016.
